# Exploring facilitators and barriers to adherence to evidence-based practice among health professionals at Tirunesh Beijing General Hospital, Addis Ababa, Ethiopia: a qualitative study

**DOI:** 10.1186/s12913-026-14923-2

**Published:** 2026-07-10

**Authors:** Abigiya Zewde Biru, Trhas Tadesse

**Affiliations:** 1 Tirunesh Beijing General Hospital, Addis Ababa, Ethiopia; 2 Yekatit 12 Hospital Medical College, Addis Ababa, Ethiopia

**Keywords:** Evidence-based practice, Adherence, Facilitators, Barriers, Qualitative study

## Abstract

**Background:**

Evidence-Based Medicine (EBM), first defined by Dr. David Sackett, is “the conscientious, explicit, and judicious use of current best evidence in making decisions about the care of individual patients.” Over time, the concept expanded to Evidence-Based Practice (EBP) to include all health professions, as outlined in the Sicily Statement, which also defines the necessary skills and educational qualifications to practice EBP effectively.

**Objective:**

To explore facilitators and barriers to adherence to Evidence-Based Practice (EBP) among health professionals at Tirunesh Beijing General Hospital, Addis Ababa, Ethiopia.

**Method:**

A qualitative phenomenological study was conducted from April to August 2024. Data were collected through 27 key informant interviews and one focus group discussion involving six health professionals selected using purposive sampling. Thematic analysis was employed to identify key themes, supported by triangulation, peer debriefing, and member checking to ensure reliability and validity.

**Results:**

Barriers to EBP adherence included administrative and systemic challenges, workforce issues, resistance to change, resource limitations, and infrastructure gaps. Facilitators included strong leadership, resource availability, accountability systems, and supportive environments. Effective implementation, monitoring, and quality improvement initiatives were also critical in promoting EBP.

**Conclusion:**

This study highlights that adherence to Evidence-Based Practice among health professionals is influenced by a combination of individual, organizational, and system-related factors. Strengthening leadership support, improving access to up-to-date clinical guidelines and resources, and enhancing continuous professional development are essential to promote optimal adherence to EBP and improve the quality of healthcare delivery.

**Supplementary Information:**

The online version contains supplementary material available at https://doi.org/10.1186/s12913-026-14923-2.

## Introduction

### Background

The two most often cited definitions of Evidence Based Medicine (EBM), which were first articulated by the late Dr. David Sackett, are as follows: “the conscientious, explicit and judicious use of current best evidence in making decisions about the care of the individual patients” [1] ,which was later refined to “EBM is a systematic approach to clinical problem solving which allows the integration of the best available research evidence with clinical expertise and patient values” [2].

Evidence-based medicine, or EBM, is a method of providing healthcare in which general practitioners base their clinical judgments on the best available evidence, or the most pertinent data, for each patient. Clinical skills, understanding of disease mechanisms and pathophysiology are all valued, enhanced, and expanded upon by EBP. It entails making intricate and careful decisions based on patient preferences, circumstances, and traits in addition to the evidence that is currently accessible. It acknowledges that health care is personalized, dynamic, and subject to both probability and uncertainty. In the end, evidence-based practice (EBM) is the formalization of the care process that generations of the best health care professionals have used. More thorough explanations of EBP have been released [3, 4].

In recent years, a number of definitions of EBP have been proposed. In order to reflect a common approach to evidence-based practice (EBP) across all health professions, the Sicily Statement [5] proposed expanding the original term “evidence-based medicine” to “evidence-based practice,” given that many health professionals have adopted an evidence-based way of practice. In addition to providing a precise definition of evidence-based practice (EBP), the Sicily Statement outlines the minimal educational and skill qualifications needed to practice EBP. This clarifies the fundamental procedures of EBP and creates a distinction between the process and the result of EBP [5].

EBP has completely changed the way medicine is done during the last thirty years. Theoretically, its core principles—management based on the Best Current Evidence, patient values and expectations, and physician skills and expertise—remain unchanged. Its practical use in the age of information explosion has, however, undergone major changes. These changes include updated definitions, broader uses of the term EBM, and improved methods for recognizing the Best Current Evidence, advancements in research and clinical application, and EBM instruction [6].

EBP is becoming more and more common across a variety of medical specialties. Its dependence on the collaboration of objective scientific data, clinical judgment, and unique patient requirements and preferences is one of its key characteristics. Because it’s critical to locate and retrieve relevant literature from a variety of sources to aid in decision-making regarding medical care, librarians have a significant impact on the dissemination of evidence-based practice (EBP) [7] (Evidence is data/information from historical or scientific evaluations of procedures that can be utilized by healthcare industry decision-makers [8].

The following five procedures comprise evidence-based practice: (1) Ask a question transforming the informational requirement into a question that can be answered, about things like therapy, diagnosis, prognosis, prevention, and causality. (2) Find information/evidence to answer question, searching for the most reliable information to address that query (3) Critically appraise the information/evidence, evaluating the evidence critically for its application (use in our clinical practice), impact (magnitude of the effect), and validity (closeness to the truth) (4) Integrate appraised evidence with own clinical expertise and patient’s preferences, combining the critical evaluation with our professional knowledge, our patients’ particular biology, values, and circumstances, and (5) Evaluate, evaluating our effectiveness and efficiency in executing Steps 1–4 and seeking ways to improve them both for next time [9].

EBP is the accepted and ideal standard for global trans-disciplinary healthcare providers, and it has the support of professional, national, and international healthcare organizations and regulatory bodies, including the National Academy of Medicine (NAM, 2022), the American Nurses Association (ANA, 2022), the Centers for Disease Control and Prevention (CDC, 2021), and the American Nurses Credentialing Center (ANCC, 2022). Reducing practice variability, improving patient outcomes, improving care quality, and lowering costs are the objectives of evidence-based decision-making (EBDM) [10].

### Statement of the problems

The amount of scientific knowledge that is now available to support medical professionals’ clinical decision-making has increased dramatically in recent years. Despite the abundance of evidence, many healthcare systems in the US and around the world still do not implement evidence-based care because of cultural norms that keep people in their “comfort zones” and a lack of competency in the EBP process among clinicians [11]. Improving patient care outcomes within the framework of intricate healthcare systems is the aim of evidence-based practice (EBP). However, the lack of daily consistency in the application of EBP by healthcare professionals makes it more difficult for healthcare organizations to provide high-quality, evidence-based care [12].

Evidence from interventional and observational studies shows persistent challenges in EBP implementation. For example, a randomized controlled trial on emergency nurses reported that while EBP education initially improved attitudes, knowledge, self-efficacy, skills, and behaviour, these effects declined after six months and returned to baseline or lower levels by 12 months [13].

Similarly, cross-sectional studies among primary care physicians in Northern Saudi Arabia and nurses in Jordan reported low to moderate levels of EBP knowledge and attitudes, and highlighted that multiple factors affecting EBP implementation involve stakeholders such as policymakers, administrators, physicians, researchers, and academics [14, 15]. In addition, findings from Peru indicated that physicians in specialty training often lack sufficient time to seek scientific evidence to answer clinical questions [16].

Consistent findings have also been reported in African settings. In Malawi, nurse-midwives demonstrated a positive attitude toward EBP but were not fully implementing it due to barriers such as lack of time, and they required improved skills in locating and appraising evidence to effectively support EBP implementation [17]. Likewise, nurse educators in Lesotho were found to lack EBP training, had difficulties formulating clinical questions, and rarely used electronic databases [18].

In Ethiopia, evidence from different regions shows similar patterns. Health care professionals in public hospitals in Buno Bedele Zone and Illu Aba Bora practiced evidence-based medicine poorly [19]. Studies from Northwest Ethiopia and South Wollo Zone further indicated that most nurses had low utilization and low adoption of EBP [20, 21]. Moreover, a systematic review and meta-analysis conducted in Ethiopia revealed that only about half (50%) of healthcare professionals practice evidence-based care effectively, highlighting the need to improve the use of current research evidence in clinical decision-making for better client care [22].

Therefore, the purpose of this study is to explore facilitators and barriers to adherence to evidence-based practice among health professionals at Tirunesh Beijing General Hospital, Addis Ababa, Ethiopia.

### Social cognitive theory (SCT)

This study is guided by Social cognitive theory (SCT), which explains human behaviour as the result of a reciprocal interaction between personal factors, environmental factors, and behaviour, ultimately influencing outcomes—in this case, adherence to evidence-based practice (EBP).

**Fig. 1:**
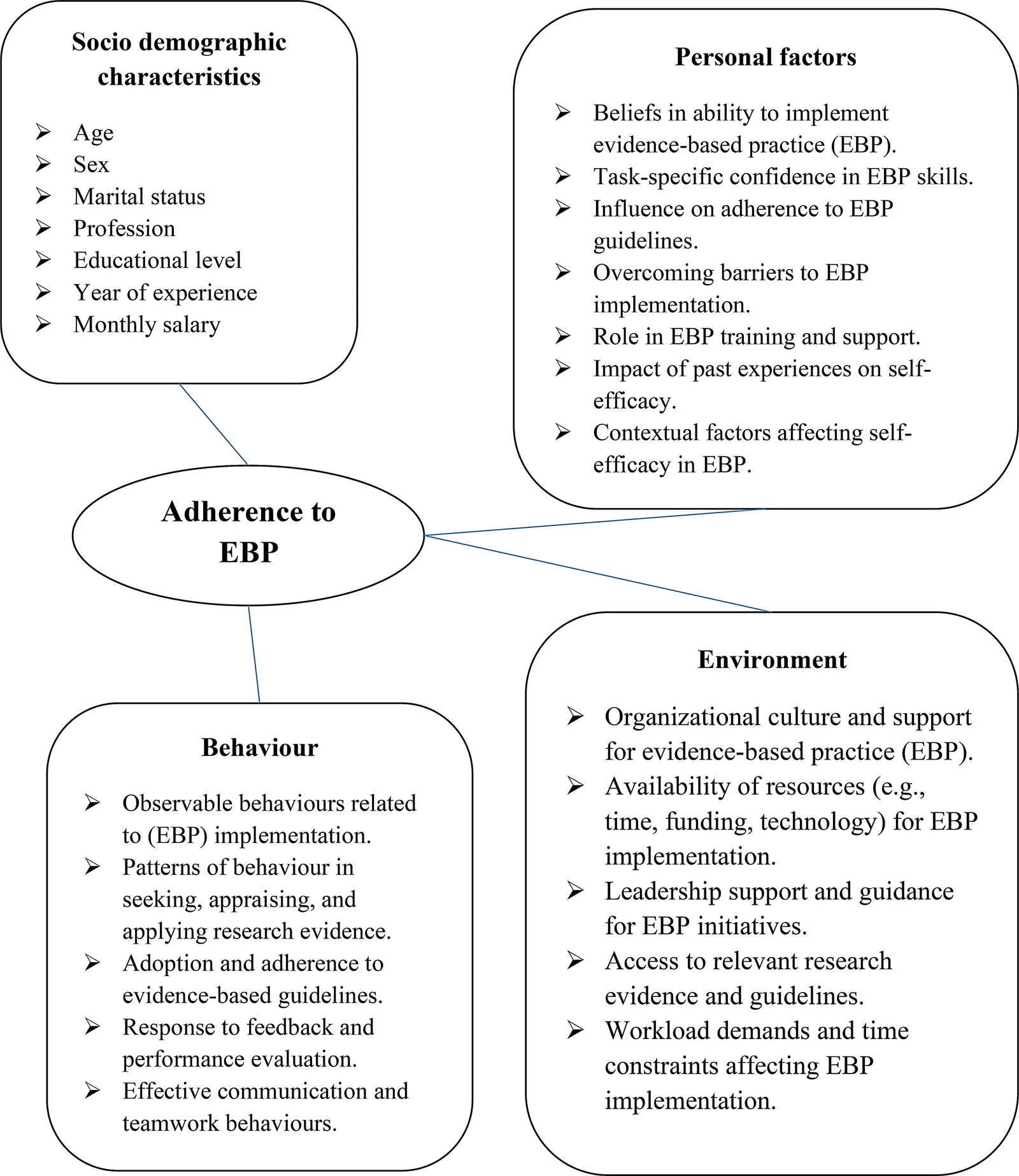
Conceptual framework adapted from Social cognitive theory [23]

Figure 1 illustrates the conceptual framework showing how personal, behavioural and environmental factors influence adherence to evidence based practice among health professionals, based on Social cognitive theory.

In the present framework, socio-demographic characteristics such as age, sex, marital status, profession, educational level, years of experience, and monthly salary are considered background factors that may influence health professionals’ personal beliefs, access to resources, and adherence to evidence-based practice.

Personal factors represent key SCT constructs, particularly self-efficacy, reflected through beliefs in the ability to adhere to EBP, task-specific confidence in EBP skills, the ability to overcome barriers, the influence of past experiences, and participation in EBP training and support. These factors shape motivation and readiness to adhere to evidence-based guidelines.

Environmental factors include organizational culture, leadership support, availability of resources (time, funding, technology), access to research evidence and guidelines, and workload demands. These elements represent the external conditions that can either facilitate or hinder adherence to EBP within the healthcare setting.

Behavioural factors refer to observable actions related to adherence to EBP, such as seeking, appraising, and applying research evidence, following evidence-based guidelines, responsiveness to feedback, and effective communication and teamwork. These behaviors reflect how personal and environmental influences translate into adherence.

Through the dynamic interaction of these components, the framework explains how adherence to evidence-based practice is achieved or constrained among health professionals at Tirunesh Beijing General Hospital. (conceptual framework adapted from Social cognitive theory [23].

## Significance of the study

Adherence to EBP is crucial in low- and middle-income country, like Ethiopia and throughout Africa, where the burden of disease is rising and there is a great need for EBP in clinical settings. In order to address the gaps in EBP adherence and remove barriers to EBP implementation, health institutions might make use of the information provided by this study. The study broadens the corpus of scientific information regarding the application of evidence-based health professional practice.

Given its critical role in patient care, the study on the adherence of evidence-based practice (EBP) and associated factors by health professionals working in Tirunesh Beijing General Hospital is important to design a strategic way to improve patient care outcomes by enhancing the application of new best evidence from research findings to the health professionals in the health sector. Government and non-government organizations involved in this field can also use the findings to inform the development of action plans and measures. Furthermore, national policies that incorporate EBP can be developed using the study’s findings and will guide health professionals’ education by identifying the areas of focus to include in the curriculum and also will serve as a baseline for other researchers conducting additional research using other research designs. In particular, it enhances patient outcomes and lowers health care costs—a goal valued highly by funding and governmental organizations.

## Objectives

### General objective

To explore the facilitators and barriers influencing adherence to evidence-based practice among health professionals at Tirunesh Beijing General Hospital, Addis Ababa, Ethiopia.

### Specific objectives

- To explore barriers to the adherence of evidence-based practice among health professionals working at Tirunesh Beijing General hospitals Addis Ababa, Ethiopia, 2024.
- To explore facilitators to the adherence of evidence base practice among health professionals working at Tirunesh Beijing General Hospital Addis Ababa, Ethiopia 2024.

## Methods

### Study area and period

The study was conducted at Tirunesh Beijing General Hospital, a public general hospital located in Addis Ababa, Ethiopia, from April 22 to August 20, 2024. Tirunesh Beijing General Hospital was constructed through collaboration between the Ethiopian and Chinese governments and has been providing comprehensive inpatient and outpatient healthcare services to the surrounding population since it became fully operational [24].

At the time of the study, the hospital employed a total of 723 healthcare professionals, including 32 public health professionals with a Master of Public Health (MPH), 64 midwives, 321 nurses, 57 general practitioners, 55 pharmacy professionals, 50 specialists, and 134 other health professionals. In addition, the hospital had 58 case team leaders from various health disciplines and was organized into 15 directorates [25].

The hospital is equipped to provide a wide range of medical services and serves a large patient population. Similar to many public hospitals in Ethiopia, Tirunesh Beijing General Hospital faces challenges related to high service demand, the growing burden of disease, emerging drug resistance, and the need to strengthen adherence to evidence-based healthcare practices [26–28].

### Study design

A qualitative phenomenological approach was employed to explore the lived experiences of health professionals regarding adherence to Evidence-Based Practice (EBP) at Tirunesh Beijing General Hospital. Phenomenology was selected because it focuses on understanding how individuals interpret and make meaning of their lived experiences, particularly in relation to complex phenomena such as EBP adherence. This approach is appropriate for capturing participants’ perceptions, meanings, and experiences regarding facilitators and barriers to EBP implementation within their routine clinical practice [29, 30]. Unlike case study designs, which typically examine a bounded system or organizational unit as a whole, phenomenology prioritizes the subjective lived experiences of individuals and how these experiences are constructed and understood. Therefore, phenomenology was considered more suitable for this study as it allowed an in-depth exploration of personal and shared experiences of health professionals in relation to EBP adherence within the hospital context.

### Population

#### Source population

The source population for this study consisted of all health professionals working at Tirunesh Beijing General Hospital, Addis Ababa, Ethiopia. This included physicians, nurses, pharmacists, laboratory professionals, health officers, and other staff involved in patient care or healthcare management within the hospital. These professionals were considered the source population because they had the potential to provide insights into adherence to evidence-based practice (EBP) in routine clinical care.

#### Study population

The study population consisted of a purposive sample of health professionals working at Tirunesh Beijing General Hospital, Addis Ababa, Ethiopia, who had direct experience with or exposure to adherence to evidence-based practice (EBP) in their routine clinical work. Participants were selected from diverse departments and professional categories to ensure a broad range of perspectives.

Eligible participants included health professionals such as physicians (general practitioners and specialists), nurses, pharmacists, laboratory professionals, health officers, and other health professionals who were actively involved in patient care and clinical decision-making and who had at least one year of work experience at the hospital. Participants were included if they had experience using, following, or being guided by evidence-based clinical guidelines, protocols, or standard treatment procedures. This experience was identified through participants’ self-reported involvement in applying clinical guidelines, participating in audit or review meetings, engaging in EBP-related discussions, or attending EBP training sessions. Data were collected using both focus group discussions (FGDs) and key informant interviews (KIIs) to capture participants’ collective experiences as well as individual insights regarding facilitators and barriers to adherence to EBP. This purposive approach ensured the inclusion of information-rich participants capable of providing meaningful qualitative data to address the study objectives.

### Eligibility criteria

#### Inclusion criteria

Participants were included if they:

- Were health professionals directly employed at Tirunesh Beijing General Hospital, Addis Ababa, Ethiopia.
- Were actively involved in patient care or clinical decision-making.
- Had direct experience with adherence to evidence-based practice (EBP), such as following clinical guidelines, participating in audits, or engaging in EBP-related discussions.
- Were willing and able to participate in focus group discussions (FGDs) or key informant interviews (KIIs).

#### Exclusion criteria

Participants were excluded if they:

1. Were administrative staff or managers not directly involved in patient care.
2. Had no prior experience with adherence to EBP.
3. Were unwilling or unable to participate in FGDs or KIIs.

### Sample size and sampling technique /sampling procedures

A total of 27 key informants (KIIs) were included, comprising department heads, team leaders, quality officers, and other professionals recognized for their expertise in evidence-based practice. Additionally, one focus group discussion (FGD) was conducted with six health professionals from various departments. The number of participants was determined based on qualitative research principles, emphasizing information richness and thematic saturation rather than statistical representativeness.

Details of the data collection methods and the study population are summarized in Table 1.

**Table 1:** Summary of data collection methods and study population

Method of data collection	Study population	Number
KII	Department heads (Directorates)	5
	Team leaders of various departments	4
	Quality officers	3
	Other individuals recognized for their expertise in evidence-based practice	15
Total (KII)		27
1 FGD	Health professionals	2
	Quality improvement coordinators	2
	Reform coordinator	1
	Member of the Research and Training Unit	1
Total (FGD)		6

### Sampling procedures

A total of 33 health professionals were selected from 723 health professionals at the hospital using purposive sampling. This method ensured the inclusion of individuals with specific expertise and roles relevant to the study’s focus.For the Key Informant Interviews (KIIs), 27 participants were chosen, including 5 department heads (Directorates), 4 team leaders from various departments, 3 quality officers, and 15 other individuals recognized for their expertise in evidence-based practice.

Additionally, 6 health professionals were selected for the Focus Group Discussion (FGD), which included 2 health professionals, 2 quality improvement coordinators, 1 reform coordinator, and 1 research professional.This sampling approach ensured the inclusion of individuals who could provide rich, detailed, and relevant insights into the research topics related to evidence-based practice.

### Data collection procedures

#### Key informant interviews (KIIs)

A total of 27 key informants were purposively selected from the source population based on their professional roles, experience, and involvement in adherence to evidence-based practice (EBP) or clinical decision-making processes at Tirunesh Beijing General Hospital. Participants included department heads, team leaders, quality officers, and other professionals recognized for their expertise in EBP. Semi-structured interview guides, developed specifically for this study and based on constructs of the Social Cognitive Theory, were used to explore facilitators and barriers to adherence to EBP. Interviews were conducted in a private setting to ensure confidentiality and were audio-recorded with participants’ consent. The recordings were transcribed verbatim for subsequent thematic analysis. This approach allowed for in-depth exploration of individual experiences and perspectives regarding EBP adherence.

#### Focus group discussion (FGD)

One Focus Group Discussion (FGD) was conducted with six purposively selected health professionals drawn from different departments to ensure diverse and information-rich perspectives on adherence to Evidence-Based Practice (EBP). Participants included physicians (general practitioners and specialists), nurses, pharmacists, laboratory professionals, health officers, and other health professionals with direct experience in implementing EBP in their routine clinical practice.

The FGD was employed to generate interactive discussions and to explore shared perceptions, norms, and collective experiences regarding facilitators and barriers to EBP adherence, which may not easily emerge in individual interviews. The group setting allowed participants to build on each other’s responses, clarify viewpoints, and highlight common challenges within the clinical environment.

Although only one FGD was conducted, this was complemented by 27 key informant interviews (KIIs), which provided in-depth individual perspectives and allowed for methodological triangulation. The combination of FGDs and KIIs enhanced data richness and contributed to thematic saturation, as no new themes emerged during analysis.

The FGD was guided by a semi-structured interview guide, conducted in a neutral and comfortable environment, audio-recorded with participants’ consent, and transcribed verbatim for analysis.

### Data quality assurance

To ensure the quality and consistency of the data collection process, careful attention was given to the preparation and use of data collection instruments. The interview and focus group discussion guides were initially developed in English and translated into Amharic to enhance clarity and cultural appropriateness.

Prior to the actual data collection, the guides were pilot-tested with health professionals at Zewditu Memorial Hospital to assess clarity, comprehension, and relevance. Feedback obtained during the pilot test was used to refine the guides.

Data were collected using audio recording to ensure completeness and accuracy. All interviews and discussions were conducted in private settings to promote openness and confidentiality. The researcher ensured consistency in conducting the KIIs and FGD by following the same procedures and using the semi-structured guides. Audio recordings were transcribed verbatim and anonymized to protect participant identity. All collected data were stored securely and accessed only by the researcher.

### Operational definitions

Evidence-Based Practice (EBP): Evidence-based practice is an approach to decision-making in which healthcare professionals integrate the best available research evidece, clinical expertise, and patient values and preferences to guide clinical practice.

Barriers to EBP: Barriers to evidence-based practice refer to factors or obstacles that hinder the implementation or adoption of evidence-based approaches in clinical practice. These barriers can include organizational factors (e.g., lack of resources, time constraints), individual factors (e.g., lack of knowledge or skills in accessing and appraising research evidence), and contextual factors (e.g., resistance to change, organizational culture).

Facilitators of EBP: Facilitators of evidence-based practice are factors or strategies that support and promote the implementation and adoption of evidence-based approaches in clinical practice. These facilitators can include organizational supports (e.g., access to resources, leadership support), educational interventions (e.g., training programs, continuing education), and professional networks (e.g., collaboration with colleagues, access to mentorship).

Adherence to EBP: Adherence to evidence-based practice refers to the degree to which healthcare professionals follow or adhere to evidence-based guidelines, protocols, or recommendations in their clinical practice. It involves consistently applying research evidence and best practices to inform clinical decision-making, treatment planning, and patient care.

### Trustworthiness of the data and researcher reflexivity

To ensure the trustworthiness of the data, several strategies were implemented. Credibility was enhanced through member checking, allowing participants to review and validate the accuracy of their contributions. Dependability was maintained by keeping an audit trail documenting all steps of the research process for transparency and replicability. Confirmability was ensured via peer debriefing, where external reviewers verified the objectivity and neutrality of interpretations. Transferability was addressed by providing a detailed description of the research context, participants, and methods, enabling readers to assess the applicability of findings to similar settings.

#### Researcher reflexivity

Researcher reflexivity was an integral part of this study and was maintained throughout data collection and analysis. The primary researcher personally conducted all interviews and focus group discussions and was actively involved in data analysis. Given the researcher’s familiarity with the clinical setting, there was a possibility that prior experiences and assumptions could influence data interpretation.

To address this, continuous reflexive practices were applied. The researcher maintained a reflexive journal to document personal reflections, assumptions, and potential biases during data collection and analysis. Bracketing techniques were used to consciously set aside pre-existing assumptions to minimize their influence on interpretation.

In addition, regular discussions were conducted within the research team, including co-authors, to critically reflect on emerging findings and interpretations. These discussions helped ensure that the analysis remained grounded in participants’ accounts rather than the researcher’s expectations or prior knowledge. Through these strategies, efforts were made to enhance transparency, reduce bias, and strengthen the credibility of the study findings.

### Data analysis procedures

The data were analyzed using thematic analysis, guided by constructs of the Social Cognitive Theory (SCT). Audio recordings from key informant interviews and the focus group discussion were transcribed verbatim to ensure accuracy and completeness. The transcripts were organized and labeled systematically for easy reference during analysis.

Data familiarization was achieved through repeated reading of the transcripts while noting initial impressions and recurring ideas. ATLAS.ti version 9.1.3.0 was used to support the coding process. Meaningful segments of text were identified and assigned initial codes, which were subsequently grouped into broader categories and themes.

Constant comparison was employed to refine and clarify themes, while analytic memos were written to document reflections and emerging insights throughout the analysis process. Themes were reviewed and refined iteratively to ensure internal consistency and clear representation of participants’ perspectives.

Peer debriefing and member checking were conducted to validate interpretations and enhance credibility. Data saturation was assessed continuously to ensure sufficient depth and completeness of the findings.

Finally, themes were interpreted in relation to Social Cognitive Theory constructs (personal, environmental, and behavioral factors) and synthesized into coherent narratives. Findings were reported using rich, descriptive language in accordance with qualitative research reporting guidelines (COREQ).

### Reporting guideline

This study followed the Consolidated Criteria for Reporting Qualitative Research (COREQ) checklist to ensure comprehensive and transparent reporting of study design, data collection, and analysis procedures [31].

### Ethical consideration

The Institutional Review Board (IRB) of Yekatit 12 Hospital Medical College (Ref. No: 571/24). College of Public Health, and Department of Health Care Quality reviewed the protocol to ensure full protection of the rights of study subjects. Following approval by the IRB, an official letter of cooperation was sent to the Addis Ababa City Adminstration Health Bureau from the Department of Health Care Quality of Yekatit 12 Medical College. After receiving permission from the Addis Ababa City Adminstration Health Bureau, a letter of cooperation was sent to Tirunesh Beijing General Hospital. As the data collector, I was informed about the study after receiving approval from the hospital. Verbal and written informed consent were obtained from study subjects. Confidentiality was assured for all the information provided, and no personal identifiers (anonymity) were used on the questionnaires.

## Result

### Socio-demographic characteristics of participants

A total of 33 participants were included in this study, comprising 27 from key informant interviews (KIIs) and 6 from a single focus group discussion (FGD). The demographic data below represent all participants. Thematic results from the FGD are presented separately in Supplementary File 1 for clarity and space considerations.

The majority of participants were male (78.8%), while females made up 21.2%. Most participants (75.7%) were aged between 31 and 40 years, with smaller proportions aged 20–30 years (18.1%) and 41–50 years (6%).Regarding educational status, a significant proportion (66.6%) of participants held a Master’s degree or higher, reflecting a highly educated cohort, while 33.3% held a Bachelor’s degree. In terms of professional experience, 48.4% had 5–10 years of work experience, and 42.4% had more than 10 years, showcasing a wealth of expertise within the sample.Professionally, nurses and midwife with a Master’s in Public Health (MPH) were the largest group (27.2%), followed by specialists (24.2%), general practitioners (18.2%), and Pharmacists with advanced degrees (15.1%). Health officers, Laboratory technologists, Anaesthetists, and midwives were less represented, with each group accounting for 3–6% of the total sample. Participants held diverse roles, with 24.2% serving as team leaders and 15.1% in directorate positions, while the majority (60.6%) were classified under other roles, reflecting a broad range of professional responsibilities.

The following table provides a detailed breakdown of the socio-demographic characteristics of all participants and is presented below as Table 2:

**Table 2:** Socio-demographic characteristics of key informant interview (KII) and focus group discussion (FGD) participants

Variable	Category	KII (*n* = 27)	FGD (*n* = 6)	Total (*n* = 33)
Gender	Male	21 (77.8%)	5 (83.3%)	26 (78.8%)
	Female	6 (22.2%)	1 (16.7%)	7(21.2%)
Profession	Specialists	7 (25.9%)	1 (16.6%)	8 (24.2%)
	General practitioners	6 (22.2%)	0 (0%)	6 (18.2%)
	MPH	6 (22.2%)	3 (50.0%)	9 (27.2%)
	Health officers	2 (7.4%)	0 (0%)	2 (6.1%)
	Pharmacists (MSc Pharmacy)	4 (14.8%)	1 (16.6%)	5 (15.1%)
	Laboratory technologists (Master’s)	1 (3.7%)	0 (0%)	1 (3%)
	Anaesthetist (MSc)	0 (0%)	1 (16.6%)	1 (3%)
	Midwife	1 (3.7%)	0 (0%)	1 (3%)
Role	Directorate	5 (18.5%)	0 (0%)	5 (15.1%)
	Team leader	5 (18.5%)	3 (50.0%)	8 (24.2%)
	Other	17 (62.9%)	3 (50.0%)	20 (60.6%)
Educational status	Degree	10 (37%)	1 (16.6%)	11 (33.3%)
	Master’s and above	17 (62.9%)	5 (83.3%)	22 (66.6%)
Age group	20–30 years	6 (22.2%)	0 (0%)	6 (18.1%)
	31–40 years	19 (70.3%)	6 (100%)	25 (75.7%)
	41–50 years	2 (7.4%)	0 (0%)	2 (6%)
Year of experience	< 5 years	3 (11.1%)	0 (0%)	3 (9%)
	5–10 years	15 (55.5%)	1 (16.6%)	16 (48.4%)
	> 10 years	9 (33.3%)	5 (83.3%)	14 (42.4%)

Notes:

KII participants: 27 key informants from different professional categories

FGD participants: 6 health professionals from diverse backgrounds

### Results of key informant interviews (KIIs)

### Domain 1: Personal factors (Knowledge, Attitudes, and Awareness of EBP) (SCT Constructs)

#### Theme 1: Understanding of evidence-based practice (EBP)

**Fig. 2:**
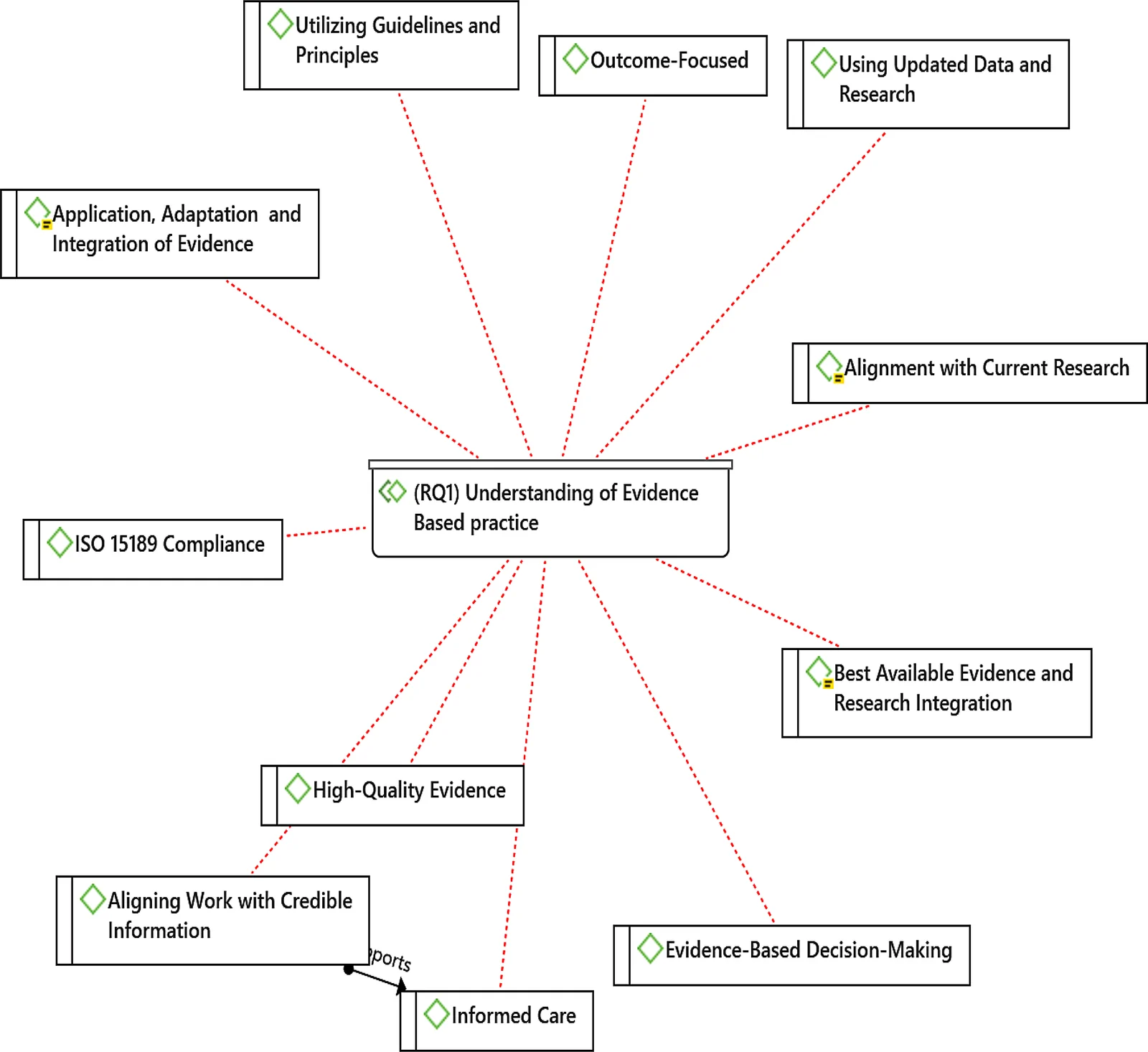
Respondents understanding of evidence-based practice

The majority of participants demonstrated a strong understanding of Evidence-Based Practice (EBP), highlighting its importance in improving patient outcomes, standardizing clinical practices, and integrating research evidence with clinical expertise. The key themes that emerged include the integration of research evidence into clinical decision-making, the adherence to established guidelines and protocols, and the consistent application of EBP across healthcare settings to enhance the quality of care (see Fig. 2).

##### Sub-theme 1.1: Definitions and personal interpretations of EBP

Most respondents defined EBP as a structured approach to healthcare, integrating clinical expertise with the best available research to ensure practices are evidence-based and aligned with standardized protocols. This understanding underlines the goal of delivering high-quality, efficient treatment while adhering to established guidelines. As one respondent noted:

Evidence-based practice combines the best available research findings, such as opinion-based studies, cohort studies, and randomized controlled trials, with clinical experience.When guidelines are established based on these studies, they provide a framework for clinical decision-making. (Respondent 15,44:1 ¶ 32).

Some participants emphasized that EBP bridges traditional practices and modern advancements by incorporating evolving knowledge into daily practice. For instance, many highlighted the transition from relying solely on physical examinations to utilizing advanced diagnostic tools, such as CT scans, as a significant improvement. One respondent shared:

In the past, we made all of our diagnosis and treatment decisions based only on physical examinations.Thanks to evidence-based practice (EBP), we can now use sophisticated instruments like CT scans, which facilitate quicker and easier diagnosis processes and enable us to act more rapidly. (Respondent 2, 31:7 ¶ 41).

Additionally, respondents stressed that EBP involves making treatment decisions supported by accurate, thoroughly researched, and reliable data. This ensures that clinical decisions are well-informed and tailored to improve patient outcomes. One participant elaborated:

When making treatment decisions for patients, medical professionals can use Evidence-Based Practice (EBP), which is the process of compiling evidence supported by many research. (Respondent 1, 30:16 ¶ 32).

Another respondent summarized the essence of EBP by highlighting its integration of research findings and clinical expertise:

It allows us to combine clinical expertise with the most recent research findings to make well-informed decisions. (Respondent 2,31:1 ¶ 32).

##### Sub-theme 1.2: General knowledge and awareness of EBP principles

The responses reflected a strong awareness of Evidence-Based Practice (EBP) principles, with participants emphasizing the importance of basing clinical decisions on solid evidence rather than arbitrary judgments. Respondents highlighted the integration of technology with clinical data and patient history as essential for ensuring precision and alignment with best practices. For instance, one participant stated:

Through the integration of these technologies with clinical data and patient history, we guarantee that our actions are precise and in line with established best practices (Respondent 2, 31:2 ¶ 32).

The responses also highlighted that respondents are aware of the need to adapt EBP principles to new challenges, including integrating new technologies and guidelines into practice. Respondents acknowledged the importance of using evolving evidence and up-to-date research to enhance clinical decisions. For instance:

EBP assists us in adjusting to changing situations, workforce availability, and resource availability. (Respondent 12, 50:41 ¶ 32).

Respondents commonly recognized the necessity of adhering to high standards and rigorous quality controls when applying EBP. These principles were described as essential for ensuring that treatments align with best practices, as well as for ensuring that the evidence supporting them has been thoroughly analyzed. Many respondents also noted that maintaining up-to-date information is crucial for effective application. For example: Respondent 12 mentioned:

By referencing established quality standards, we align our practices with evidence-based guidelines, ensuring effective and reliable patient care (Respondent 12,49:34 ¶ 32).

There was a consensus among respondents regarding the importance of having a broad understanding of EBP principles. Most recognized that EBP is not static but requires adapting to changing circumstances such as shifts in guidelines, available resources, or evolving health conditions. One respondent mentioned how healthcare professionals must remain flexible in applying EBP:

EBP assists us in adapting to changing circumstances, such as fluctuating labor availability, resources, and circumstances. (Respondent 13, 41 ¶ 32).

Additionally, respondents pointed out the need for adhering to both international and local guidelines in clinical decision-making. However, they acknowledged the challenges of potential inconsistencies when different practitioners follow different guidelines. One respondent expressed concern over this issue:

If some practitioners adhere to U.S. guidelines while others follow national or regional guidelines, inconsistencies may arise in treatment protocols. (Respondent 7, 36:44 ¶ 55).

##### Sub-theme 1.3: Perceived importance of EBP in clinical practice

Respondents strongly agreed on the perceived importance of Evidence-Based Practice (EBP) in clinical practice. The majority emphasized that EBP is not only integral to improving treatment outcomes but also crucial for enhancing patient care and ensuring optimal healthcare delivery. Respondent 7 noted that EBP’s integration of high-quality research into clinical practice is fundamental in improving care:

EBP enhances the quality of care and ensures continuous improvement in healthcare delivery (Respondent 7, 36:27 ¶ 44).

The perceived value of EBP was also expressed in terms of its role in adapting to evolving healthcare challenges. Respondents pointed out that staying current with evidence not only ensures better decision-making but also facilitates the application of new knowledge to address contemporary health issues. Respondent 12 elaborated on the role of EBP in adapting to medical advancements:

Adapting to the latest evidence and procedures because medical practice is continuously advancing (Respondent 12, 50:17 ¶ 46).

Moreover, the majority of the respondents linked EBP to the continuous improvement of healthcare interventions, noting that by relying on up-to-date evidence, clinicians can ensure that their practices remain effective and efficient. Respondent 6 highlighted the importance of applying reliable evidence:

In this globalized age, when people have access to a wealth of knowledge, I think it’s essential to comprehend and use it wisely. (Respondent 6,35:12 ¶ 43).

**Fig. 3:**
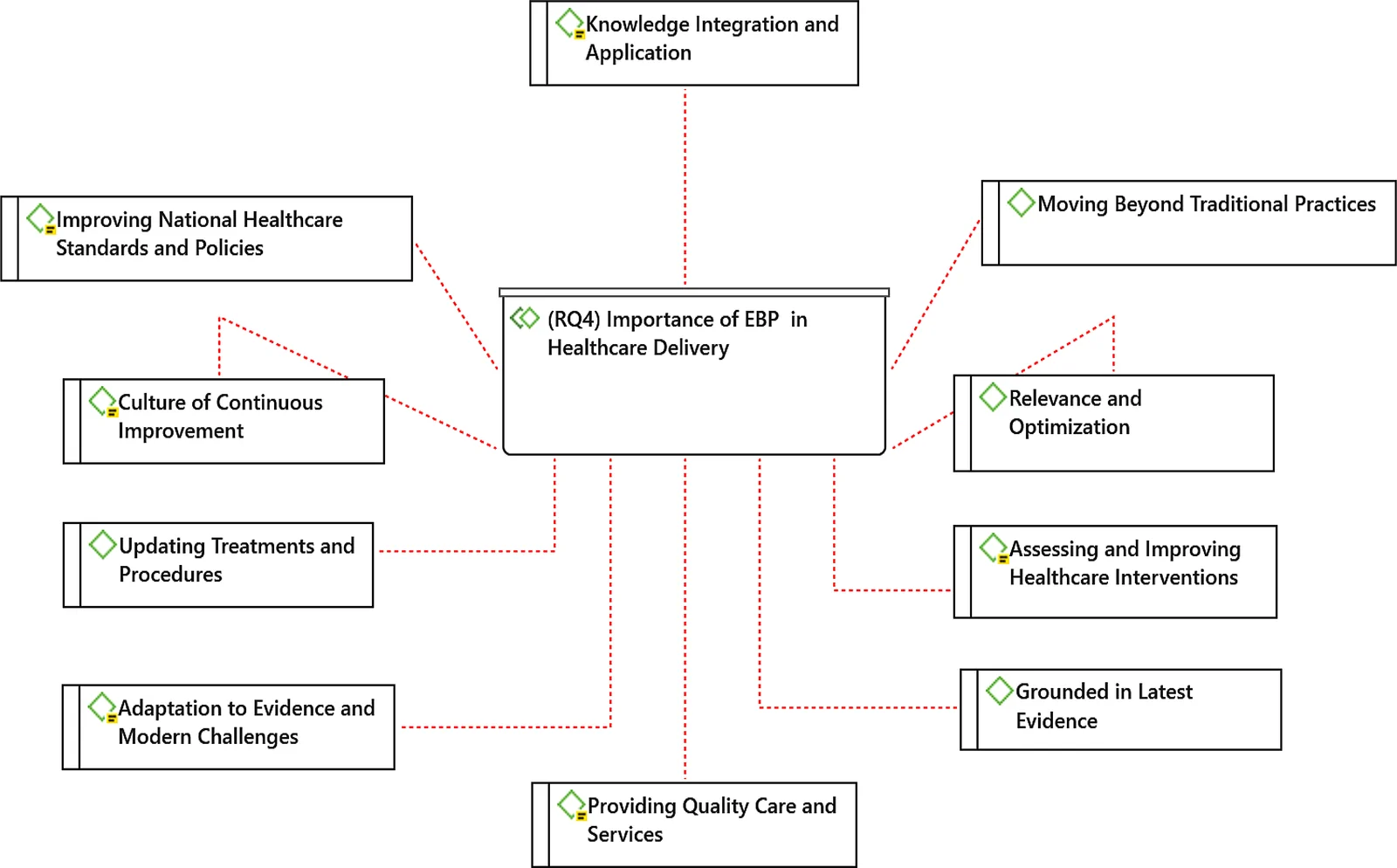
Importance of EBP in health care delivery with connected codes

The majority of respondents emphasized the essential role of Evidence-Based Practice (EBP) in healthcare delivery. Most of them highlighted that EBP is critical to clinical practice because it ensures that services and treatments are based on the best available evidence, which ultimately enhances the quality of care and patient outcomes. Respondents noted that continuous learning and the acquisition of practical skills are fundamental to successfully integrating EBP into clinical settings. A key point made was that having access to up-to-date information and staying informed through regular training opportunities were crucial for maintaining adherence to EBP (see Fig. 3). For example, Respondent 6 stated:

Access to up-to-date information, continuous learning, and skill development are important factors that facilitate adherence to evidence-based practice at Tirunesh Beijing General Hospital. (Respondent 6:)

### Domain 2: Environmental/contextual and personal barriers to EBP (SCT Constructs)

#### Theme 2: Challenges and barriers to adherence to EBP

Challenges refer to situational or contextual difficulties experienced by healthcare professionals in adhering to EBP (e.g., workload, time constraints, hierarchical culture).

Barriers refer to structural or systemic factors that hinder adherence, such as lack of resources, insufficient leadership support, or absence of training programs.

The majority of respondents identified various challenges in adherence to Evidence-Based Practice (EBP), focusing primarily on issues related to access to resources, resistance to change among staff, and workload challenges. These barriers significantly impact the adoption of EBP, leading to inconsistencies in patient care and hindering the effectiveness of clinical decision-making.

Adherence to Evidence-Based Practice (EBP) faces numerous challenges that hinder its full integration into healthcare settings. These barriers exist at individual, institutional, and systemic levels, limiting the consistent application of EBP principles in clinical practice.

##### Sub-theme 2.1: Individual-level barriers (lack of knowledge, negative attitudes)

Individual-level barriers such as knowledge gaps, resistance to change, and negative attitudes significantly hinder the adherence to Evidence-Based Practice (EBP) among healthcare professionals. Many practitioners lack a thorough understanding of EBP or perceive it as an additional burden rather than an integral aspect of their responsibilities. These barriers are further compounded by ingrained habits and insufficient motivation.

##### Knowledge gaps and resistance to change

Several respondents emphasized the lack of adequate education and awareness about EBP as a primary challenge. Some practitioners do not perceive gathering and utilizing evidence as part of their clinical duties, leading to inconsistent application. For instance, one respondent noted:

If healthcare professionals do not view gathering and using information as part of their work, they may be less inclined to adopt EBP in their clinical practice (Respondent 6, 35:24).

Reliance on traditional practices and expert opinions was also highlighted as a barrier:

Reliance on expert opinion can also be problematic (Respondent 7, 36:38).

Without adequate knowledge and training, healthcare professionals struggle to integrate EBP into their routines, viewing it as an optional rather than essential component of patient care.

##### Negative attitudes and lack of motivation

Negative attitudes towards EBP were another recurring theme. Many practitioners resist change, often due to a perception that EBP disrupts established workflows or adds to their workload. As one respondent shared:

Some staff may not prioritize EBP, seeing it as less important than other daily duties (Respondent 6, ¶55).

This resistance is exacerbated by a lack of encouragement and support from leadership:

Without adequate motivation and encouragement from leadership, health professionals may struggle to engage with EBP initiatives (Respondent 6, ¶56).

A positive attitude was noted as a key enabler of EBP adoption. However, in its absence, ingrained habits and skepticism towards new practices impede progress:

A positive attitude toward EBP makes its adoption smoother, but without it, professionals may resist (Respondent 7).

##### Administrative challenges and systemic impacts

Respondents also pointed out that administrative resistance to new information could hinder the integration of EBP into clinical settings:

Administrative acceptance of new information is one of the biggest challenges health professionals faces when implementing EBP (Respondent 7, 36:33 ¶50).

This attitude can hinder the integration of new practices and make it more difficult for EBP to become fully embedded in clinical settings (Respondent 7, 36:40 ¶52).

**Fig. 4:**
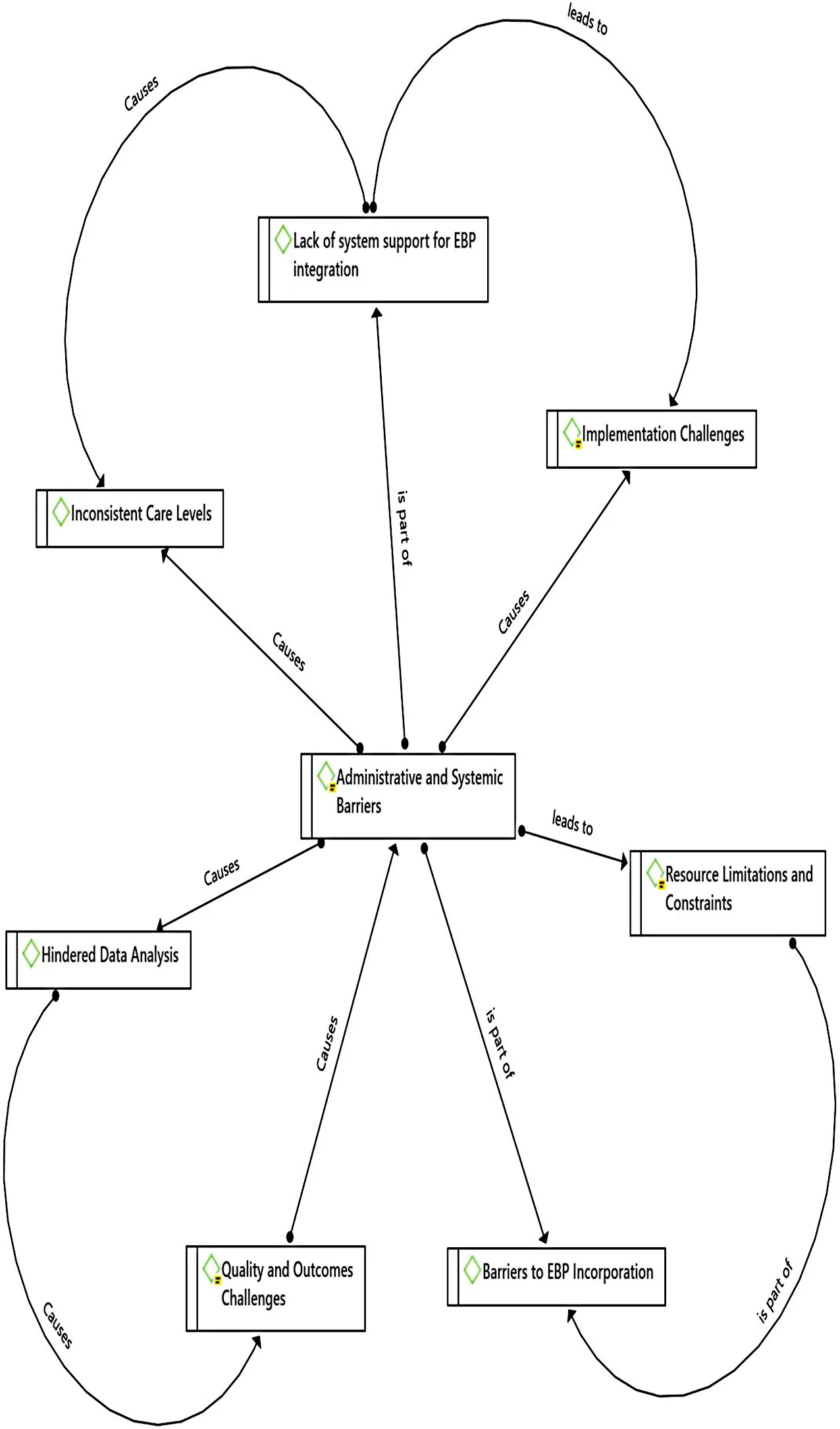
Administrative and systemic barriers to EBP adherence

These challenges are further illustrated in Fig. 4, which presents a network analysis of administrative and systemic barriers to EBP adherence.

Staff resistance to change was a recurrent theme. This resistance often results from a lack of knowledge about EBP principles, inadequate training, or a reluctance to embrace new practices that might interfere with long-standing habits.

I’ve been doing things this way for years; why change what seems to work? (Respondent 5: 36:33 ¶ 50).

Without a unified policy or protocol from national policymakers, the variation in protocols between hospitals creates resistance among staff (Respondent 15: ¶57).

Resource limitations, including insufficient training for staff, are a significant obstacle to consistent EBP implementation (Respondent 14: ¶52).

Resistance to adopting new practices was another key barrier. Many respondents noted that healthcare professionals often rely on previous experiences, creating reluctance to incorporate newer, evidence-based approaches.

Challenges arise when senior professionals rely solely on expert opinion, disregarding newer, evidence-based updates. (Respondent 7, 36:18 ¶ 39).

We worked without clear direction—essentially, acting ‘without a clue,’ as staff were hesitant to embrace structured EBP methods. (Respondent 9, 49:16 ¶ 38).

Resistance to change is often rooted in attitudes, which can hinder integration of new practices. (Respondent 14, 36:40 ¶ 52).

Time constraints and workload challenges significantly impede the integration of EBP. Healthcare professionals often cite heavy patient loads and competing responsibilities as barriers to engaging with EBP initiatives, leading to inconsistent application.

Applying Evidence-Based Practice (EBP) can be challenging due to variations in how it is implemented and the time needed for thorough application (Respondent 3, ¶41).

There are instances where certain practices may not be feasible due to time constraints and the pressure of daily workloads (Respondent 13, ¶49).

This neglect can lead to serious consequences for patient care, undermining the goals of EBP” (Respondent 9, ¶54).

I barely have time to see my patients, let alone look up new evidence or attend training sessions. (Respondent 7).

##### Sub-theme 2.2: Institutional barriers (lack of infrastructure, funding)

Institutional barriers such as inadequate infrastructure, insufficient funding, and limited organizational support were frequently cited by respondents as significant challenges to implementing Evidence-Based Practice (EBP). These systemic issues hinder healthcare professionals’ ability to consistently adopt evidence-based approaches, affecting both the quality and efficiency of care delivery.

##### Infrastructure limitations

Inadequate infrastructure, including limited access to diagnostic tools, internet connectivity, and physical resources, emerged as a prominent barrier. Respondents highlighted the unavailability of essential diagnostic equipment and modern technologies as a significant hindrance to EBP implementation:

The absence of sufficient diagnostic resources presents a major obstacle to the implementation of evidence-based practice (EBP) (Respondent 1, 30:20 ¶40).

The availability of modern diagnostic equipment, especially MRI machines, presents a significant barrier for medical professionals practicing evidence-based practice (Respondent 2, 31:18 ¶56).

Access to updated guidelines is another critical issue. Without internet connectivity or hard copies of guidelines, healthcare professionals struggle to stay informed about best practices:

The absence of hard copies further emphasizes the need for internet connectivity (Respondent 12, ¶52).

Infrastructure challenges extend to incomplete data management systems, which impede decision-making:

Data compilation is vital for decision-making, but incomplete registration and lack of infrastructure impair this process (Respondent 10, ¶68).

##### Financial constraints

Budget limitations were widely reported as a critical barrier to EBP. These constraints impact the procurement of diagnostic tools, implementation of training programs, and hiring of adequate staff:

Budget constraints can also pose a significant barrier (Respondent 12, 51:30 ¶70).

Additionally, insufficient funding hampers the ability to maintain or upgrade infrastructure, further compounding the challenges faced by healthcare providers.

##### Workforce challenges

Heavy workloads and inadequate staff-to-patient ratios were frequently cited as obstacles to prioritizing EBP. High demands on healthcare professionals limit their time and capacity to engage in evidence-based practices:

Inadequate staff-to-patient ratios and heavy workloads make it challenging to prioritize EBP (Respondent 4).

A lack of ongoing training exacerbates the issue. Without continuous education and skill development, healthcare professionals struggle to stay updated on evidence-based guidelines:

Insufficient ongoing training can impede the effective application of EBP, as healthcare professionals need continuous education to stay updated with best practices (Respondent 3).

##### Organizational and leadership gaps

Respondents also identified limited organizational support as a key barrier. Poor incentives, lack of recognition, and minimal accountability diminish motivation to adopt EBP:

Incentives, acknowledgment, and accountability are lacking, which may have a negative effect on engagement and motivation (Respondent 1: ).

Limited access to evidence or resources is a significant barrier to the effective implementation of EBP. Many healthcare professionals expressed difficulties in obtaining current and relevant research due to insufficient resources. Respondents emphasized the need for advanced tools and resources to support EBP, while also acknowledging the limitations in their availability.

The absence of sufficient diagnostic resources presents a major obstacle in implementing evidence-based practices (Respondent 1:30:20 ¶ 40).

The availability of modern diagnostic equipment, especially MRI machines, is limited, affecting our ability to apply EBP efficiently (Respondent 2: 31:18 ¶ 56).

There’s just not enough access to the latest studies; we often rely on outdated information because we can’t find the evidence we need.

Improving the hospital’s system setup is crucial to support evidence-based practice effectively. (Respondent:32:30 ¶ 68).

Several respondents highlighted that limited access to resources and outdated diagnostic tools make it challenging to adhere to evidence-based practices (EBP). Issues such as equipment shortages, power outages, and insufficient staffing were recurrent themes.

The availability of resources is a key barrier to adhering to evidence-based practice, especially given budgetary constraints and economic issues. (Respondent 2: 31:23 ¶ 64).

Frequent power outages disrupt EMR, limiting our ability to use EBP fully. (Respondent 7: 35:20 ¶ 54).

Improving the hospital’s system setup is crucial to support evidence-based practice effectively. (Respondent 3: 32:30 ¶ 68).

Proper data management was identified as a cornerstone of EBP, yet respondents cited difficulties with data collection, tracking, and decision-making. Inadequate monitoring of treatment outcomes, such as death rates in emergency rooms, led to less-informed decision-making.

One of the significant challenges in implementing EBP is improper data collection, which undermines the reliability of treatment decisions. (Respondent 10, 51:17 ¶ 39).

We faced issues tracking sudden death rates due to inadequate data collection, affecting our ability to create effective indicators. (Respondent 10: 51:35 ¶ 39).

Data-driven frameworks are essential for decision-making processes, yet they are often not fully established in practice. (Respondent 15: 51:6 ¶ 32).

Several respondents expressed concerns over the quality of evidence used in clinical settings. Distinguishing between high- and low-quality studies was mentioned as a challenge that could directly impact patient care outcomes.

The inability to differentiate between high- and low-quality studies creates further challenges, risking the application of poor-quality research. (Respondent 7: 36:21 ¶ 39).

In settings where resource limitations are prevalent, implementing EBP can be difficult and may not yield the desired outcomes. (Respondent 14: 43:8 ¶ 41).

##### Sub-theme 2.3: Cultural or systemic resistance to change

Resistance to adopting Evidence-Based Practice (EBP) is often rooted in both cultural and systemic factors, creating significant barriers to its implementation. These challenges stem from deeply ingrained beliefs, traditional practices, and organizational inconsistencies that hinder the seamless integration of evidence-based approaches.

##### Cultural resistance to change

Cultural norms and attitudes within healthcare institutions significantly influence EBP adoption. Respondents emphasized that longstanding reliance on traditional practices, particularly among senior professionals, creates resistance to updating care methods:

Resistance to change is a significant obstacle, as implementing EBP necessitates modifying long-standing practices (Respondent 1).

Senior professionals may sometimes believe that their experience outweighs the importance of current evidence (Respondent 7).

There is a tendency among professionals to adhere to traditional practices rather than adopting new evidence-based approaches (Respondent 3).

This cultural reluctance is further complicated by differences in local practices compared to global standards. Such cultural resistance delays the acceptance of EBP, particularly in settings where traditional practices are valued over emerging evidence. For example:

The treatments or guidelines used in places like America might not align with our system (Respondent 15, 44:24).

##### Systemic barriers

Systemic challenges, including inconsistent policies, frequent management changes, and a lack of standardized guidelines, further impede EBP implementation. Respondents highlighted the absence of a unified framework for information exchange as a critical barrier:

Without a unified policy or protocol from national policymakers, the variations in information exchange among institutions can lead to inconsistencies in care (Respondent 15, 44:32 ¶57).

Additionally, with changes in management, the updating of standard treatment protocols is often delayed (Respondent 13, 42:26).

Frequent shifts in leadership disrupt continuity, making it challenging to sustain EBP initiatives. Moreover, inconsistent policies across institutions create confusion and hinder the alignment of practices with evidence-based guidelines.

##### Interplay between cultural and systemic barriers

Cultural and systemic resistance often interact, amplifying the challenges to EBP adoption. For instance, the reluctance of senior staff to embrace change may discourage management from enforcing updated protocols, further delaying EBP integration. Similarly, systemic delays in providing clear guidelines can reinforce reliance on traditional practices, creating a cycle of stagnation.

**Fig. 5:**
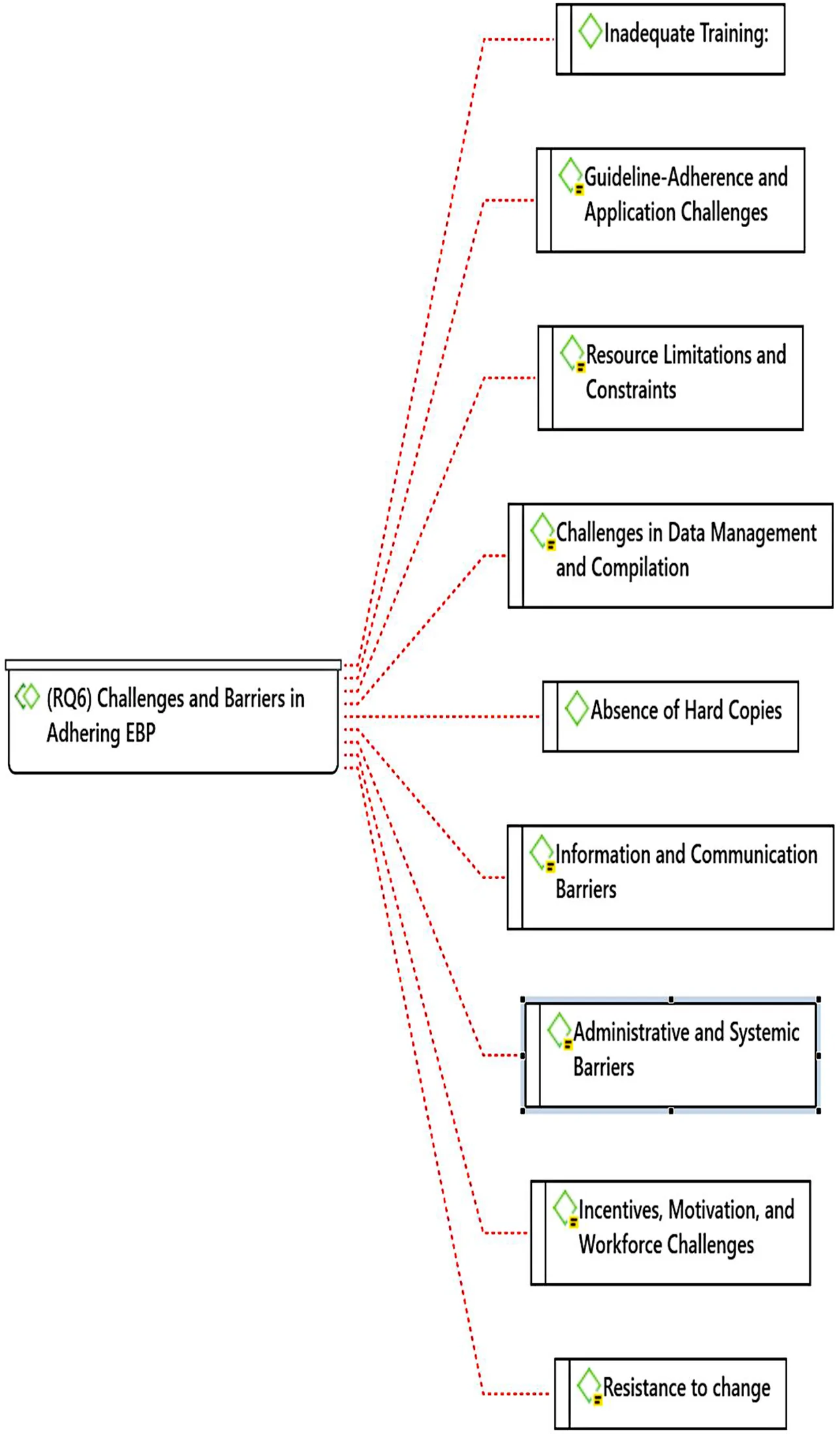
Challenges and barriers to adherence to EBP

These overlapping challenges are illustrated in Fig. 5, which presents a summary of barriers to adherence to Evidence-Based Practice.

### Domain 3: Positive reinforcement and self-ffficacy in EBP (SCT Constructs)

#### Theme 3: Success stories in adherence to EBP

Evidence-Based Practice (EBP) is recognized by respondents as a transformative approach that significantly improves patient outcomes and healthcare delivery. Respondents emphasized that EBP enhances clinical practices, reduces mortality rates, improves decision-making, and ensures consistent, error-free care.

##### Sub-theme 3.1: Positive outcomes from EBP adherence (improved patient care, efficiency)

Respondents highlighted that EBP has significantly improved patient care and efficiency in healthcare delivery. Effective adherence to EBP was reported to lead to better patient outcomes, streamlined processes, and overall enhanced quality of care. Respondents shared how EBP supports precise diagnoses, consistency in treatment, and resource efficiency:

Despite these challenges, EBP has been successful in transforming patient care and improving quality. When effectively adhered, EBP leads to better patient outcomes and enhanced healthcare delivery, demonstrating its value in advancing clinical practice. (Respondent 3: 32:10 ¶42).

We have seen significant benefits from integrating EBP into our processes, leading to better patient care and enhanced decision-making. (Respondent 9: 49:36 ¶ 40).

It is an essential part of providing efficient, quality healthcare. (Respondent 1: 30:27 ¶ 46).

We guarantee more precise diagnoses and improved patient outcomes. (Respondent 2: 31:13 ¶ 48).

Respondents also shared specific examples:

Since we adopted evidence-based protocols, our patient care has significantly improved, leading to a noticeable reduction in complications (Respondent 1).

Another respondent emphasized the efficiency gained, stating,

Implementing EBP has streamlined our processes and helped us use resources more effectively, which ultimately benefits our patients (Respondent 3).

##### Sub-theme 3.2: Iinterdisciplinary collaboration

Respondents noted that EBP fosters interdisciplinary teamwork, enabling healthcare professionals from various fields to collaborate effectively. This teamwork helps standardize care and integrate diverse perspectives, ultimately improving healthcare delivery.

Experience sharing and ongoing training play significant roles in implementing EBP across departments. (Respondent 9).

Handled complex cases through experience sharing, showing that interdisciplinary support is essential for successful application. (Respondent 12).

On the successful side, we’ve managed to handle complex cases through collaboration and discussion around evidence-based best practices (Respondent 12).

A positive attitude towards EBP was also seen as crucial for fostering interdisciplinary collaboration.

Therefore, fostering a positive attitude towards EBP is essential for enhancing interdisciplinary collaboration. (Respondent 9: 49:1 ¶ 54).

An open and adaptive attitude towards EBP is crucial for effectively integrating interdisciplinary collaboration. (Respondent 3: 32:25 ¶ 63).

Ultimately, evidence-based practice is a matter of life and death, guiding healthcare professionals in their interdisciplinary roles to provide better care. (Respondent 13: 42:14 ¶43).

Specific examples highlighted the benefits of collaboration:

Working together as a team—doctors, nurses, and pharmacists—has allowed us to integrate various perspectives and improve our care plans (Respondent 2).

Our interdisciplinary meetings have fostered better communication and teamwork, which have been crucial for applying the best available evidence in patient care (Respondent 4).

##### Sub-theme 3.3: Decision-making through EBP

Respondents agreed that EBP enhances healthcare decision-making by providing thoroughly researched, evidence-based guidelines. This approach minimizes reliance on outdated methods or intuition, resulting in more precise and efficient clinical decisions.

EBP allows us to improve the quality of our services, leading to better patient care and enhanced decision-making. Respondent 9 (49:13 ¶ 40).

Regularly conducting clinical audits to evaluate adherence to evidence-based practice ensures better decision-making and patient outcomes. Respondent 14 (43:5 ¶ 37).

We guarantee more precise diagnoses and improved patient outcomes, which result from better clinical decision-making based on EBP. Respondent 2 (31:5 ¶ 36).

Respondents highlighted the reduction of variability and adherence to clinical guidelines as key benefits.

With EBP, we rely on high-quality research rather than just our experience, which has improved the effectiveness of our interventions (Respondent 5).

The shift towards evidence-based decision-making has reduced variability in our practices and improved adherence to clinical guidelines (Respondent 6).

Moreover, EBP was recognized as critical for patient safety and quality improvement:

“Guidelines create opportunities for effective treatment,” emphasizing that evidence-based decision-making directly impacts patient outcomes. (Respondent 13)

Ultimately, evidence-based practice is a matter of life and death, guiding us to make the best possible choices for patient care (Respondent 13).

### Domain 4: Environmental and personal facilitators of EBP (SCT Constructs)

#### Theme 4: Factors facilitating adherence to EBP

Adherence to Evidence-Based Practice (EBP) is driven by factors such as resource availability, leadership support, and continuous training. These elements create an environment where healthcare professionals can consistently apply updated knowledge in their practice. Institutional backing, professional development opportunities, and individual motivation were highlighted as critical drivers for sustained adherence and meaningful improvements in clinical care.

##### Sub-theme 4.1: Clear protocols and institutional support

Strong institutional support was repeatedly identified as essential for EBP adherence. Leadership commitment, resource provision, and fostering a culture of accountability were highlighted as critical components. Respondents noted that effective leaders set positive examples, allocate necessary resources, and create an environment conducive to evidence-based practices.

For instance, the Ministry of Health’s initiatives, such as SBFR and EHAQ, provide structured guidance and resources to sustain EBP. Hospital administrators also play a vital role by ensuring the availability of essential medical devices, even amidst resource shortages.

The Ministry of Health’s initiatives, like the SBFR, EHAQ, and EBC programs, offer great assistance and direction… guaranteeing that EBP will continue to be developed and implemented. (Respondent 1, ¶49).

The management is responsible for assuring the supply of vital medical devices, which are frequently unavailable, and understands their importance. (Respondent 2, ¶52).

A culture of engaged leadership and collaboration encourages accountability and ensures that resources and support systems are consistently available to maintain high EBP standards.

Encourage leaders at all levels to champion EBP initiatives, ensuring that they allocate resources and support necessary for successful implementation. (Respondent 10, ¶ 95).

The administrative side also plays an important role by supporting these efforts. (Respondent 15, ¶ 46).

A prominent strategy discussed by respondents is the development and adherence to clear protocols and guidelines that standardize healthcare practices and ensure consistency in the delivery of care. Most respondents highlighted that structured and regularly updated guidelines contribute significantly to successful EBP adherence. For instance, Respondent 1 shared:

We have outlined the appropriate treatment for patients with specific conditions in our clearly defined scope of practice for physicians. This entails creating detailed protocols for every disease entity and making sure they are implemented in clinical settings. (Respondent 1, 30:18 ¶ 36).

This practice is particularly emphasized in departments like nursing, where creating and executing guidelines that promote evidence-based care is a focal point. Respondent 7 added:

Administratively, promoting EBP can be supported by clear guidelines and protocols that encourage adherence. (Respondent 7, 36:12 ¶ 35).

##### Sub-theme 4.2: Integration of technology and resources

Another vital strategy identified by the respondents is the integration of advanced technologies to facilitate and support EBP. This includes using evidence-based technologies to ensure the collection of accurate data and the application of best practices. Respondent 1 explained the use of technological integration:

These standards are developed through extensive trials, expert opinions, and the integration of these technologies with clinical data to enhance patient care. (Respondent 3, 32:3 ¶ 32).

Some respondents pointed to the integration of advanced diagnostic technologies and tools as a major factor in supporting EBP. They stressed that the use of cutting-edge tools such as CT scans, online databases, and diagnostic platforms enhances clinical decisions and patient outcomes. This combination of technology with clinical expertise forms the backbone of modern EBP approaches.

Integrating advanced diagnostic technologies like CT scans with clinical knowledge is one effective neurosurgical approach that supports evidence-based practice (Respondent 2, 31:4 ¶ 36).

Additionally, the implementation of international standards like ISO 15,189 was recognized as crucial for maintaining high-quality practices, with Respondent 9 stating:

Implementing ISO 15189 standards have proven effective in ensuring quality results. (Respondent 9: 49:37 ¶ 35).

Lastly, EMR (Electronic Medical Recording) systems represent a key technological tool that supports EBP by making patient data more accessible, accurate, and consistent across different departments. The integration of EHRs enables healthcare providers to align their practices with the latest evidence and clinical guidelines by providing real-time access to patient histories, lab results, and treatment plans. This helps Equipping Staff to Maintain Standards.

Ensuring that our team is well-informed and equipped to maintain these high standards is essential, especially when integrating new technologies such as EHRs. (Respondent 9, ¶ 35).

##### Sub-theme 4.3: Culture of continuous learning and motivation

Continuous professional development (CPD) emerged as a crucial factor in maintaining adherence to EBP. Respondents frequently cited the importance of specialized training sessions, workshops, and bedside audits to reinforce EBP principles. CPD opportunities ensure healthcare staff stay informed about the latest EBP guidelines and practices, creating a foundation for effective implementation.

Providing continuous professional development (CPD) training for clinical staff, ensuring they stay updated with EBP principles. (Respondent 3, ¶37).

Training opportunities such as clinical audits, morning sessions and rounds activities help ensure that healthcare staff stay informed about the latest EBP guidelines and practices.

Conducting clinical audits and reviewing patient charts help ensure that practices are aligned with EBP guidelines. (Respondent 3, ¶ 51).

Regular updates, such as interdisciplinary rounds and the sharing of new guidelines, also play a significant role. Respondents noted that multidisciplinary approaches create enriched learning environments that foster collaboration and reinforce adherence to EBP.

We share and normalize updated guidelines that we’ve developed in clinical rounds, helping staff stay current with EBP standards. (Respondent 12, ¶ 35).

Multidisciplinary approach strengthens adherence to evidence-based practices across departments. (Respondent 12, ¶ 49

Respondents stressed the importance of creating a culture of continuous education and improvement as an essential factor for fostering EBP. Regular seminars, research discussions, and workshops help update healthcare professionals on the latest evidence, ensuring that practices remain current. Respondent 12 shared:

Regular morning and seminar sessions, experience sharing, and discussions around evidence-based best practices help align our approach with the latest research. (Respondent 12: 50:24 ¶ 49).

The necessity of ongoing education and professional development to advance EBP was a recurrent subject among those surveyed. It was stated that creating a culture that promotes lifelong learning, skill improvement, and involvement in research is crucial. According to the respondents, cultivating such a culture improves healthcare professionals’ capacity to apply EBP successfully and keeps them up to date on the most recent research.

Fostering a culture of continuous improvement in healthcare (Respondent 9: 49:21 ¶ 45).

Individual commitment and motivation are critical to integrating EBP into daily practice. A supportive environment that empowers and recognizes healthcare professionals was noted as a key factor in promoting adherence to EBP. Respondents emphasized that personal dedication to EBP enhances patient care, job satisfaction, and proactive engagement with evidence-based decisions.

When healthcare professionals collectively accept the significance of such evidence, it encourages a proactive approach to data management. (Respondent 10, ¶76).

The success of evidence-based practice (EBP) is clear in how it helps transform patient care and achieves favourable outcomes. (Respondent 6, ¶ 38).

**Fig. 6:**
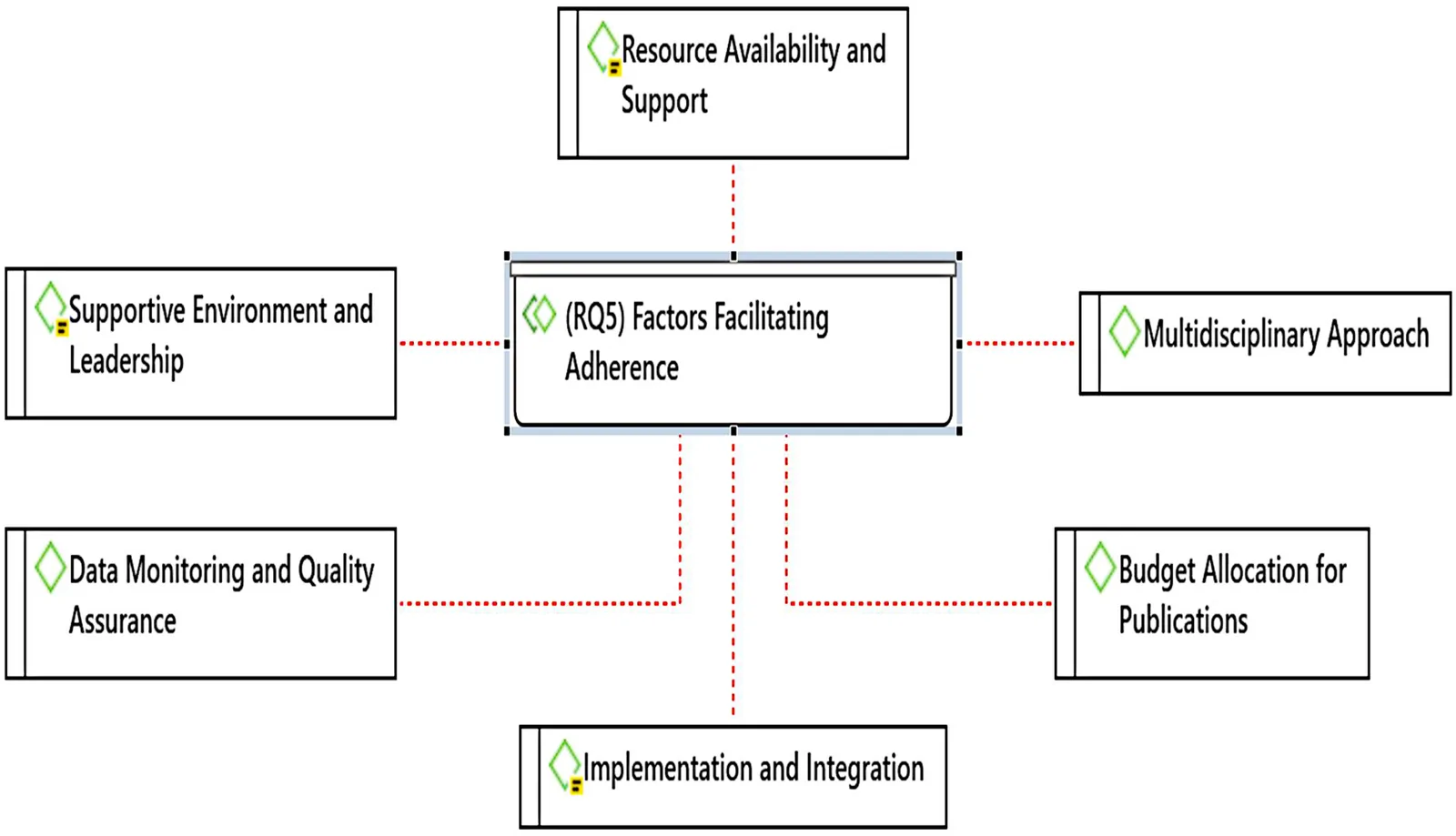
Respondents response for facilitators of Evidence Based Practice adherence

A collaborative culture where evidence is created and shared reinforces individual commitment to EBP. Respondents highlighted the role of senior staff in championing EBP and inspiring broader adherence across departments (see Fig. 6). 

Active involvement of senior staff in promoting and practicing EBP encourages broader adherence and integration throughout the hospital. (Respondent 3, ¶ 54).

Encourages staff to actively engage in evidence creation and application. (Respondent 12, ¶ 49).

#### Theme 5: Recommendations for improving EBP adherence

##### Sub-theme 5.1: Suggestions for better training and education

Respondents emphasized the importance of continuous training and education to keep healthcare professionals updated on the latest evidence-based practices (EBP). Rapid advancements in medicine necessitate ongoing learning to avoid reliance on outdated practices. Respondent 2 highlighted this by stating:

Given how quickly medicine is evolving, it’s important to acknowledge that procedures performed ten years ago might not be applicable today. To align with EBP, we need to constantly update our knowledge and procedures.

Respondents recommended holding frequent, focused training sessions to help close the gap between theoretical understanding and real-world application. These were considered essential for fostering competence and self-assurance in medical personnel. Important findings include:

Changing attitudes and enhancing knowledge through regular updates and training can improve adherence to EBP (Respondent 3, ¶52).

Ongoing awareness and education are essential; regular training sessions keep staff informed about the latest practices (Respondent 4, ¶46).

Providing practical training opportunities allows healthcare professionals to apply EBP principles in real time (Respondent 6, ¶47).

Practical recommendations included expanding training on digital literacy, improving access to EBP resources, and facilitating peer-to-peer learning. For instance, Respondent 6 noted:

Facilitating experience exchange among professionals supports the effective implementation of EBP.

Respondents also emphasized the importance of collaborative forums, such as seminars and annual meetings, to promote a culture of continuous learning. Respondent 12 suggested:

Promoting collaboration through seminars, meetings, and shared journals can establish a culture of continuous improvement.

However, a lack of digital proficiency among staff was identified as a barrier to accessing and utilizing EBP resources effectively. Respondent 6 explained:

A lack of proficiency in computer use and software applications prevents staff from accessing and utilizing electronic resources.

Developing competence and confidence in clinical decision-making through EBP was seen as a cornerstone of improving patient care. Respondents noted:

Understanding the importance of EBP ensures that clinical decisions are based on the most recent research (Respondent 1, ¶55).

A positive attitude toward EBP is crucial for effective clinical decision-making (Respondent 4, ¶52).

##### Sub-theme 5.2: Need for policy changes or updated guidelines

Many respondents called for updated organizational policies and standardized guidelines that integrate current evidence into routine care. They stressed the importance of aligning national and institutional protocols to improve adherence to EBP. Respondent 1 recommended:

Effective implementation of initiatives like Ethiopian Hospital Alliance for Quality (EHAQ) and Evidence-Based Care (EBC) is crucial to increase adherence to EBP.

Clear and regularly updated policies were seen as essential for maintaining consistency in practice. Respondent 14 suggested:

Encouraging healthcare professionals to read and understand guidelines, such as the Ethiopian Hospital Service Improvement Guideline (EHSIG) and EBC protocols, enhances knowledge and commitment to EBP.

Respondents also identified the need for a centralized platform to provide easy access to updated guidelines, research publications, and protocols. For example:

To achieve a standardized national framework, collaboration among healthcare stakeholders is vital to create comprehensive and evidence-based guidelines (Respondent 15, ¶44:33).

Providing easy access to journals and research publications empowers health professionals to stay informed and engaged with EBP (Respondent 15, ¶44:41).

The lack of accessible platforms for obtaining manuals and guidelines was seen as a significant barrier. Respondent 13 noted:

There is a lack of accessible platforms for obtaining manuals at every location.

Additionally, respondents suggested tailoring national guidelines to local needs by incorporating hospital-specific quality improvement (QI) projects. Respondent 1 explained:

By adding hospital-specific QI projects to national guidelines, we have successfully created and executed local protocols.

Respondents emphasized that frequent updates to policies and guidelines are necessary to keep pace with changes in evidence and practice. Respondent 13 remarked:

If changes occur frequently or rapidly, prompt updates are essential.

##### Sub-theme 5.3: Recommendations for improving leadership and support systems

One of the most important suggestions for developing an Evidence-Based Practice (EBP) culture was to improve organizational support and leadership. The necessity of mentorship, active training engagement, and leadership that values cooperation and knowledge exchange was often emphasized by respondents. It was believed that leaders play a key role in fostering an atmosphere that supports EBP and inspires staff members to pursue ongoing education and skill improvement.

##### Promoting a culture of learning and collaboration

Leadership was frequently described as the cornerstone for establishing a culture of continuous learning. Respondents emphasized the importance of fostering teamwork, open communication, and ongoing development. Leaders are expected to encourage health professionals to adapt to advancements in healthcare practices and actively support EBP adherence.

“Fostering an environment that prioritizes continuous learning and adaptation” is vital to make EBP more engaging and practical (Respondent 7: ¶36:54).

“Health professionals must cultivate patience and a willingness to share their EBP knowledge with colleagues,” underscoring the role of leaders in building a collaborative environment (Respondent 14: ¶43:22).

Respondent 9 advocated for

“Fostering a culture of continuous improvement in healthcare” as a sustainable approach to integrating EBP into everyday practice (Respondent 9: ¶49:21).

##### Leadership-driven support systems

The active involvement of leaders in implementing and sustaining EBP was recognized as fundamental. Respondents recommended establishing dedicated teams to regularly update guidelines and disseminate information effectively. They also stressed the importance of utilizing communication tools to enhance the accessibility of EBP resources.

The hospital effectively motivates adherence to evidence-based practice (EBP) by utilizing its own communication system to disseminate new information via platforms like Telegram, noted Respondent 13 (¶42:15).

To ensure timely updates, there should be a dedicated team responsible for this task, added Respondent 13 (¶42:27).

##### Addressing systemic challenges

Several respondents pointed out systemic barriers, such as resource constraints, excessive workloads, and outdated systems, that hinder healthcare professionals’ ability to implement EBP effectively. Leadership was seen as crucial in addressing these challenges to create an enabling environment for evidence-based care.

Respondent 4 recommended addressing staffing levels, stating,

Improving the nurse-to-patient ratio allows healthcare professionals to focus more on implementing EBP without being overwhelmed by excessive workloads (¶69).

Respondent 3 highlighted the need for better system design, suggesting that hospitals must have

Functional and user-friendly systems that facilitate evidence-based care (¶79).

Respondent 3 further emphasized overcoming resource constraints, stating,

Address and overcome resource constraints to ensure that necessary tools and support are available for effective EBP implementation (¶78).

##### Mentorship and knowledge sharing

Respondents highlighted the importance of mentorship programs and professional experience exchanges to reinforce EBP principles. Leadership was urged to prioritize mentorship as a means of improving staff confidence and competence in EBP application.

“Learning about these developments is crucial as traditional medicine moves toward evidence-based practice,” noted Respondent 2, emphasizing the role of leaders in bridging the knowledge gap (¶36:12).

Respondent 12 suggested that fostering collaboration among professionals through “seminars, annual meetings, and forums for sharing journals and publications

Could promote a culture of continuous learning and improvement (¶50:32).

## Discussion

This study explored the facilitators and barriers to adherence to evidence-based practice (EBP) among healthcare professionals at Tirunesh Beijing Hospital. The findings highlight a complex interplay of individual, institutional, and systemic factors that either enhance or hinder adherence to EBP in clinical practice. Healthcare professionals identified several key facilitators, including continuous training, institutional support, personal motivation, and a collaborative environment. Conversely, barriers such as knowledge gaps, negative attitudes, inadequate resources, and cultural or systemic resistance impeded consistent adherence. Understanding these factors is essential for designing strategies that support sustained adherence to evidence-based guidelines, ultimately improving patient care.

### Facilitators of EBP

#### Continuous professional development and structured training

A major facilitator identified was the availability of continuous professional development (CPD) and structured training for healthcare staff. Respondents emphasized that regular training sessions, clinical audits, and sharing updated guidelines during clinical rounds ensured practices remained aligned with EBP standards. This finding is consistent with previous literature, which highlights CPD, online support guides, and ongoing communication as key facilitators of EBP [32–34]. Structured discussions and electronic education modules, for example, have been shown to support adherence to evidence-based guidelines in stroke rehabilitation and long-term care [32, 33].

The present study further demonstrates that, within the Ethiopian healthcare context, training contributes not only to knowledge acquisition but also to increased professional confidence and motivation to apply evidence consistently in clinical practice. This suggests that adherence to EBP depends not only on awareness of evidence-based recommendations but also on healthcare professionals’ perceived competence and confidence in applying them. These findings align with Social Cognitive Theory (SCT), which emphasizes the importance of self-efficacy in influencing professional behavior and sustained adherence to recommended practices.

#### Institutional support and resource availability

Institutional support and resource availability also emerged as important facilitators of EBP adherence. Participants described leadership commitment, provision of medical equipment, and access to updated guidelines as essential factors enabling healthcare professionals to adhere to evidence-based recommendations. Similar findings have been reported in previous studies, where management support and organizational commitment were identified as critical components for promoting a culture of evidence-based care [32–35].

In the context of Tirunesh Beijing General Hospital, where resource limitations remain common, participants repeatedly emphasized that supportive leadership and adequate supplies enabled healthcare professionals to consistently follow recommended clinical protocols. This finding highlights the importance of organizational readiness and environmental support in sustaining adherence to EBP. According to SCT, environmental facilitation significantly influences behavior, indicating that healthcare professionals are more likely to adhere to evidence-based guidelines when institutions provide the necessary infrastructure, resources, and administrative support.

#### Individual motivation and commitment

Individual motivation and professional commitment were also identified as key facilitators of adherence to EBP. Participants described how healthcare professionals who recognized the value of evidence-based care were more likely to engage actively in guideline adherence, share knowledge with colleagues, and advocate for evidence-based interventions. These findings are consistent with previous literature identifying positive attitudes, motivation, and self-efficacy as important determinants of EBP adherence [36, 37].

The findings of this study suggest that motivated healthcare professionals are more likely to develop a sense of responsibility toward maintaining evidence-based standards in clinical practice. This reinforces SCT concepts related to self-regulation and behavioral reinforcement, where individuals who perceive positive outcomes from adherence are more likely to sustain such behaviors over time.

#### Collaborative environment and team-based learning

Collaboration and interdisciplinary teamwork were also reported as important facilitators of EBP adherence. Participants highlighted that knowledge sharing, peer support, and interdisciplinary discussions encouraged healthcare professionals to integrate evidence into routine practice. Previous studies similarly report that collaborative work environments and team-based learning approaches strengthen adherence to evidence-based guidelines [33, 38, 39].

In this study, the involvement of senior staff and experienced professionals in mentoring and supporting junior staff contributed to broader acceptance of evidence-based protocols. This finding reflects the SCT concept of social modeling, whereby observing peers and leaders practicing EBP reinforces adherence behaviors among other healthcare professionals. Therefore, collaborative learning environments may play a critical role in strengthening organizational culture and promoting sustainable adherence to EBP.

### Barriers to EBP

#### Individual-level barriers

Despite the identified facilitators, several individual-level barriers hindered adherence to EBP. Participants frequently mentioned inadequate knowledge, limited awareness of updated guidelines, negative attitudes toward EBP, and resistance to change, particularly among some senior professionals. Similar barriers have been reported in previous studies examining adherence to evidence-based guidelines among healthcare workers [32, 34, 36, 40].

Some participants perceived EBP as time-consuming or as an additional responsibility beyond routine clinical duties. This perception may reduce healthcare professionals’ willingness to consistently follow evidence-based recommendations. Furthermore, insufficient confidence in interpreting and applying research evidence limited adherence among some participants. These findings suggest that limited self-efficacy and unfavorable outcome expectations may negatively influence adherence behaviors, consistent with SCT principles.

The findings indicate the need for targeted educational interventions, mentorship programs, and supportive supervision to improve healthcare professionals’ confidence and attitudes toward EBP. Strengthening motivation and recognition systems may also enhance sustained adherence.

#### Institutional and systemic barriers

Institutional and systemic barriers were also prominent challenges affecting adherence to EBP. Participants reported shortages of medical supplies, inadequate infrastructure, limited access to updated clinical guidelines, staffing shortages, and insufficient training opportunities as significant obstacles. Similar findings have been documented in previous studies conducted in resource-constrained healthcare settings [32, 35, 41, 42].

Heavy workload and inadequate staff-to-patient ratios were frequently mentioned as barriers limiting healthcare professionals’ ability to consistently apply evidence-based recommendations. In addition, inadequate incentives and lack of recognition discouraged participation in EBP activities, particularly in high-pressure clinical environments [33, 43]. Organizational challenges such as inconsistent leadership support, policy instability, and lack of standardized procedures further contributed to poor adherence [44–46].

Cultural and hierarchical factors also influenced adherence behaviors. Some participants reported that traditional practices and reliance on senior professionals’ opinions were sometimes prioritized over research-based recommendations. Similar findings have been reported in studies examining organizational culture and resistance to evidence-based change [39, 40, 43]. These findings suggest that adherence to EBP is influenced not only by individual competence but also by broader organizational and systemic conditions.

Overall, the findings support SCT assumptions that professional behavior is shaped through interactions between individual beliefs, environmental conditions, and organizational systems. Therefore, improving adherence to EBP requires comprehensive strategies addressing both personal and institutional barriers.

### Implications

#### Policy implications

The findings suggest that healthcare institutions and policymakers should prioritize strategies that strengthen adherence to EBP. Institutional policies should promote continuous professional development, improve access to updated clinical guidelines, and ensure adequate resource allocation to support adherence. Leadership commitment and supportive supervision are also essential for creating healthcare environments that encourage consistent adherence to evidence-based standards.

#### Theoretical implications

This study highlights the relevance of Social Cognitive Theory in understanding adherence to EBP among healthcare professionals. The findings demonstrate that individual factors such as motivation, self-efficacy, and attitudes interact with environmental and organizational conditions to influence adherence behaviors. This supports the SCT perspective that professional behavior is shaped through reciprocal interactions between personal, behavioral, and environmental factors.

#### Research implications

Future research should focus on evaluating interventions designed to improve adherence to EBP among healthcare professionals. Studies examining the effectiveness of structured training programs, mentorship systems, interdisciplinary collaboration, and organizational support mechanisms may provide important evidence for strengthening adherence. Further research exploring context-specific barriers in resource-limited settings is also recommended.

### Limitation and strength

#### Limitations

This study has several limitations. First, the study was conducted in a single hospital setting, which may limit the transferability of the findings to other healthcare institutions with different organizational structures, resources, and clinical contexts. Second, although the sample size was adequate for a qualitative phenomenological study, inclusion of participants from additional healthcare facilities may have provided broader perspectives on adherence to Evidence-Based Practice (EBP). Third, as with other qualitative studies, the interpretative nature of thematic analysis involves a degree of researcher subjectivity. To enhance rigor, reflexivity was maintained throughout data collection and analysis, and continuous peer discussions were used to reduce potential bias and strengthen credibility. Additionally, conducting interviews within a busy hospital environment created scheduling challenges, which may have limited the time available for some interviews.

#### Strengths

This study has several strengths. The qualitative phenomenological design enabled an in-depth exploration of healthcare professionals’ lived experiences, perceptions, and attitudes regarding adherence to Evidence-Based Practice (EBP). The use of both key informant interviews (KIIs) and focus group discussions (FGDs) enhanced the richness and comprehensiveness of the data by capturing both individual and group perspectives. Methodological triangulation strengthened the credibility and trustworthiness of the findings. In addition, systematic data management using ATLAS.ti supported structured coding and rigorous thematic analysis. Furthermore, the study provides valuable context-specific insights into facilitators and barriers of EBP adherence within a resource-limited Ethiopian hospital setting, which may inform similar healthcare contexts.

## Conclusion

This study explored facilitators and barriers influencing adherence to evidence-based practice (EBP) among health professionals at Tirunesh Beijing General Hospital in Addis Ababa, Ethiopia. The findings demonstrate that adherence to EBP is shaped by the interaction of individual, organizational, and system-level factors; with contextual and structural conditions playing a key role in clinical practice.

Overall, improving adherence to EBP requires strengthening professional competencies while simultaneously addressing systemic and organizational barriers that limit the application of evidence in routine care. Creating a supportive work environment, improving resource availability, and reducing workload pressures are essential for sustaining evidence-based practice. Future research should focus on evaluating integrated interventions targeting multi-level determinants of EBP adherence in similar settings.

### Recommendation

Based on the findings, the following recommendations are proposed to improve adherence to Evidence-Based Practice (EBP) among healthcare professionals.

#### Institutional strengthening

Health facilities should integrate EBP into routine clinical practice by developing clear guidelines, protocols, and structured frameworks that support consistent evidence-based decision-making.

#### Leadership and governance

Hospital leadership should actively promote a culture of EBP through commitment, resource allocation, and continuous advocacy to ensure that evidence-based care is prioritized at all levels.

#### Capacity building

Continuous and targeted training programs should be implemented to improve healthcare professionals’ skills in critical appraisal, application of evidence, and updating clinical knowledge based on current research.

#### Health information systems

EBP resources and clinical guidelines should be integrated into hospital electronic systems, including EMR platforms, to improve accessibility and support real-time clinical decision-making.

#### Workforce and resources

Improving staffing levels and ensuring availability of essential medical equipment and diagnostic tools is necessary to reduce workload pressures and facilitate adherence to EBP.

#### Organizational culture and collaboration

Hospitals should promote continuous learning through regular clinical audits, seminars, and interdisciplinary collaboration to strengthen knowledge sharing and EBP adoption.

#### Behavioral and cultural change

Efforts should be made to reduce reliance on traditional or opinion-based practices by promoting awareness of the benefits of EBP, including improved patient outcomes and efficiency.

#### Motivation and incentives

Recognition and incentive mechanisms should be introduced to encourage healthcare professionals who consistently apply EBP in clinical practice.

#### Future research

Future studies should examine the long-term impact of structured EBP interventions and explore context-specific barriers in different healthcare settings. Quantitative and mixed-methods studies are recommended to further measure determinants of EBP adherence.

## Supplementary Information

Below is the link to the electronic supplementary material.


Supplementary Material 1



Supplementary Material 2


## Data Availability

The datasets generated and/or analyzed during this study are available from the corresponding author upon reasonable request.

## Abbreviations

ANCC: American Nurses Credentialing Center
ANA: American Nurses Association
CDC: Centers for Disease Control and Prevention
CPGs: Clinical Practice Guidelines
EBC: Evidence–Based Care
EBDM: Evidence–Based Decision–Making
EBM: Evidence–Based Medicine
EBP: Evidence–Based Practice
EBTs: Evidence–Based Treatments
EHAQ: Ethiopian Hospital Alliance for Quality
FGD: Focus Group Discussion
KII: Key Informant Interview
MDT: Multidisciplinary Team
NAM: National Academy of Medicine
TBGH: Tirunesh Beijing General Hospital
WHO: World Health Organization
