# Supplementary material for: Exploring facilitators and barriers to adherence to evidence-based practice among health professionals at Tirunesh Beijing General Hospital, Addis Ababa, Ethiopia: a qualitative study
Source: BMC Health Serv Res. 2026 Jul 10;26:1194. doi: 10.1186/s12913-026-14923-2 (PMC13523195; doi:10.1186/s12913-026-14923-2)
Supplement: Supplementary file 1 [file 12913_2026_14923_MOESM1_ESM.docx]

# Results of Focus Group Discussions (FGDs)

# Theme 1: Understanding and Incorporating EBP

## **Sub-theme 1.1: Definition and Understanding**

Respondents emphasized that Evidence-Based Practice (EBP) represents a science-driven approach to healthcare, contrasting with traditional methods. They noted that EBP is grounded in scientific rigor, integrating current research findings into clinical practice:

"In a health facility, EBP is essentially a science-based treatment approach, meaning that our practices should be driven by scientific evidence rather than traditional methods" (FGD Data: 1:52 ¶51).

Most respondents described EBP as an approach that uses reliable, research-backed information to guide clinical decisions, which they viewed as crucial for improving healthcare quality and patient outcomes. They highlighted EBP as a systematic framework requiring updated, high-quality evidence to address clinical challenges:

"We identify problems that could impact our services and propose possible solutions, guided by high standards of research and evidence" (FGD Data: ¶89).

Additionally, respondents highlighted EBP's role as a policy framework:

"EBP itself functions as a policy, providing a framework for practice" (FGD Data: ¶172).

Additionally, several respondents defined EBP as a professional approach to healthcare that integrates the best available evidence, clinical expertise, and patient values, ensuring high-quality care and improved patient outcomes.

“EBP is about integrating various forms of knowledge and skills to deliver effective care” (FGD Data: 1:79 ¶79).

## **Sub-theme 1.2: Incorporating EBP into Work**

Respondents provided insights into how EBP is practically integrated into their roles. Many described the use of standard operating procedures (SOPs), protocols, and checklists to standardize practices based on current evidence:

"We incorporate EBP by developing documents such as standard operating procedures (SOPs) and guidelines" (FGD Data: 1:61 ¶61).

Departments actively identify and update necessary protocols to align with best practices:

"Using checklists, we assess whether departments are following the appropriate standards, and if gaps are found, we address them by introducing the necessary guidelines and protocols" (FGD Data: 1:63 ¶61).

"As these protocols are updated, they are continuously revised to replace outdated practices, helping to maintain a current approach" (FGD Data: 1:81 ¶81).

Respondents also emphasized the importance of ongoing research to support EBP. For instance, one participant noted their institution's involvement in a research conference with 79 researchers studying COVID-19:

"For example, our institution participated in a research conference where 79 researchers were involved in studying COVID-19" (FGD Data: 1:68 ¶68).

Research findings, bulletins, and recent journals are made accessible to staff to inform clinical practice:

"We make research findings, bulletins, journals, and other recent materials available to the hospital community" (FGD Data: 1:75 ¶74).

Clinical discussions and literature reviews further promote a shared understanding of EBP among staff:

"Over the past two years, these discussions and literature reviews have contributed to making our practices more evidence-based" (FGD Data: 1:47 ¶41).

Despite progress, respondents highlighted challenges in incorporating EBP into daily work, including resistance to change, lack of training, and a preference for familiar practices:

"Implementing EBP can be challenging because many people tend to stick to familiar practices, and there is often resistance to adopting new evidence" (FGD Data: ¶75).

To address these barriers, respondents noted several strategies:

Community Awareness:

"We incorporate EBP by drawing from our recent experiences. For example, we create community awareness about the hospital’s services by sharing successful practices and highlighting past mistakes where evidence wasn’t followed so others can learn from them" (FGD Data: 1:74 ¶74).

**Capacity Building:**

Continuous training and education were cited as essential to improving knowledge and adherence to EBP:

"One important factor that facilitates adherence to EBP is capacity-building efforts such as training and education to improve knowledge and understanding" (FGD Data: ¶214).

**Monitoring and Evaluation**:

Regular monitoring and evaluation were deemed critical for the successful application of protocols and guidelines:

"To ensure that protocols and EBP guidelines are applied successfully, routine monitoring and evaluation are necessary" (FGD Data: ¶121).

**Scope-Based Practice:**

Respondents highlighted the importance of professionals operating within their defined scopes of practice:

"Professionals are now able to operate within their specific scopes of practice, ensuring that the right services are delivered by those with the appropriate skills and educational preparation" (FGD Data: 1:5 ¶12).

# Theme 2: Impact of Evidence-Based Practice

In analyzing the responses, the majority of participants emphasized the transformative impact of Evidence-Based Practice (EBP) on improving clinical outcomes and modernizing healthcare delivery. They consistently highlighted that EBP enables healthcare providers to make informed, patient-centered, and data-driven decisions, leading to enhanced service quality and improved patient care.

## **Sub-theme 2.1: Positive Experiences**

Participants shared several examples of how EBP has significantly improved practices within their departments:

a. **Quality Improvement and Hypothermia Reduction**

One respondent described a quality improvement initiative in the Neonatal ICU aimed at preventing hypothermia in preterm babies. By wrapping preterm infants in polythene plastic during transport, hypothermia cases were reduced by over 50%. This intervention demonstrated the power of implementing research findings in practice.

"Research showed that wrapping preterm babies in polythene plastic immediately during transport can reduce hypothermia by 50%. We implemented this finding, and as a result, we reduced hypothermia cases by over 50%" (FGD Data, ¶32).

b. **Viral Suppression and ART Improvement**

EBP also played a pivotal role in improving outcomes for patients on antiretroviral therapy (ART). Initially, viral suppression rates were between 80-85%. However, after implementing EBP-based quality improvement methods, the rate increased to over 90%.

"Using quality improvement methods, we increased the number of patients tested, achieving over 90% viral suppression" (FGD Data, ¶33).

c. **Enhanced Patient Outcomes Through Standards Application**

Many respondents noted that EBP helped identify gaps in clinical practices. Applying established standards allowed teams to determine whether tasks were completed, identify reasons for non-completion, and address areas needing improvement.

"When these standards are applied, they help us identify gaps, whether tasks have been completed, why they may have been neglected, and where improvements are needed" (FGD Data, ¶37).

d. **Modernized Medical Care**

Participants also highlighted the broader impact of EBP in fostering a research-based approach to care. By integrating updated evidence into daily practices, healthcare providers ensured high-quality service delivery.

"These discussions promote a research-based approach to care, ensuring every practice is rooted in high-quality evidence" (FGD Data, ¶26-27).

Summary of Impact

The majority of participants agreed that implementing EBP has greatly enhanced clinical procedures, management efficiency, and patient outcomes. They emphasized that EBP leads to structured decision-making processes, improved service quality, and the adoption of up-to-date standards, resulting in consistent and superior patient care.

Regular team discussions, literature reviews, and clinical meetings were cited as essential tools for fostering EBP adoption. As one participant reflected:

"Over the past two years, these discussions and literature reviews have significantly helped us adopt EBP, improving our clinical management and services" (FGD Data, ¶41).

Participants also highlighted the role of expert-led sessions in promoting best practices. Senior professionals guided teams through research findings and their practical applications:

"An important practice we’ve adopted is incorporating expert opinions. Senior professionals or experts lead morning sessions where different teams come together to discuss and process information based on research findings" (FGD Data, ¶26).

Use of Standardized Documents and Research Integration

Standardized tools, such as checklists, were identified as critical for maintaining high-quality care across departments. These documents ensured protocols were followed systematically and helped identify areas needing improvement:

"Incorporating EBP through standardized documents like checklists helps us ensure that every department follows protocols and assesses its practices systematically. This approach has been crucial in maintaining high-quality care" (FGD Data, ¶61).

Additionally, integrating findings from research conferences into daily practice was seen as valuable for fostering a culture of continuous learning and improvement:

"For example, our institution participated in a research conference, and we found that integrating the findings into our daily practice was valuable. It contributed to a stronger culture of ongoing learning and improvement" (FGD Data, ¶68).

By adopting EBP, participants reported substantial improvements in clinical management, patient outcomes, and the overall modernization of healthcare practices within their institutions.

# Theme 3: Facilitators of EBP Adherence……

The majority of respondents identified several critical factors that support adherence to Evidence-Based Practice (EBP). These facilitators were seen as essential for encouraging healthcare professionals to consistently implement and sustain EBP in their daily practices. Key facilitators included structured monitoring and accountability systems, access to accurate information, fostering ownership among practitioners, implementation of clear frameworks, demonstration of positive outcomes, and the availability of financial and resource support.

## **Sub-theme 3.1: Factors Supporting Adherence**

**Monitoring and Support**

Structured monitoring systems and regular performance evaluations were frequently emphasized as vital for ensuring adherence to EBP. Respondents noted that these processes help identify gaps and ensure compliance with established guidelines, protocols, and standard operating procedures (SOPs). One participant stated:

“The Ministry of Health actively monitors and supports this initiative, ensuring its continuous improvement and implementation.” (FGD DATA: 1:88)

**Access to Accurate Information**

Healthcare professionals emphasized the importance of reliable information and clear procedures to enable effective implementation of EBP. Accurate resources were seen as critical for ensuring tasks are performed efficiently and align with evidence-based standards. As one respondent noted:

“Importantly, the users of EBP themselves need the right information and procedures, which helps them execute their tasks effectively and ensures the initiative’s progress.” (FGD DATA: 1:92)

**Accountability Systems**

Accountability was highlighted as a cornerstone for promoting sustained adherence to EBP. Respondents stressed the need for systems that address deficiencies and hold individuals or departments accountable for lagging performance. These systems foster a sense of responsibility and encourage consistent improvement. As one participant explained:

“Regular performance monitoring teams can help track EBP implementation, identify gaps, and explore areas for improvement.” (FGD DATA: 1:123)

**Fostering Ownership and Team Accountability**

Many respondents noted that team-based approaches to monitoring promote ownership and accountability. Involving practitioners in these processes motivates them to take responsibility for their roles in implementing EBP, thus contributing to the sustainability of the practice. One participant shared:

“This team-based approach fosters a sense of ownership and accountability, making it easier to sustain EBP practices.” (FGD DATA: 1:164)

**Structured Frameworks**

The implementation of clear frameworks, such as Evidence-Based Care (EBC), System Bottleneck Focus Reform (SBFR), and the Ethiopian Hospital Service Improvement Guideline (EHSIG), was identified as a key enabler for EBP adherence. These frameworks provide structured guidelines that ensure consistency in practice. Respondents also highlighted the role of research departments and continuous training in equipping healthcare professionals with the skills to apply EBP effectively.

“Several initiatives promote EBP, such as EBC, SBFR, and EHSIG, and the existence of these initiatives plays a significant role in encouraging the practice of EBP.” (FGD DATA: 1:112 ¶ 12)

**Positive Outcomes as a Motivator**

The visible impact of EBP on patient care served as a powerful motivator for healthcare professionals to sustain its practice. Many participants reported that observing improvements in patient outcomes reinforced their commitment to applying EBP. One respondent remarked:

“When we implement a practice and see positive results, such as patient improvement, it motivates us to continue applying EBP.” (FGD DATA: 1:129 ¶ 137)

**Financial and Resource Support**

The availability of adequate resources, tools, and funding was frequently mentioned as crucial for effective EBP implementation. Respondents emphasized that financial incentives, such as funding for research and evidence application, play a significant role in promoting adherence. One participant noted:

“Strong financial backing plays a crucial role in promoting EBP. For instance, financial incentives to bring evidence and apply it in practice can motivate us to adopt EBP more consistently.” (FGD DATA: 1:128 ¶ 136)

Another respondent elaborated:

“Ensure robust financial backing for EBP initiatives. Adequate funding is essential to provide the necessary resources, tools, and infrastructure to support EBP practices effectively.” (FGD DATA: 1:139 ¶ 212)

The facilitators identified are key enablers of adherence to Evidence-Based Practice, supporting both individual and institutional commitment. These interrelated factors are illustrated **in Figure 7**.

Figure 7: Facilitators and Linkages to EBP Adherence

# Theme 4: Barriers to EBP Adherence

## **Sub-theme 4.1: Leadership and Management Barriers**

Respondents highlighted gaps in leadership and management as significant obstacles to EBP adherence. Limited engagement from leaders, insufficient motivation, and weak accountability systems were seen as primary barriers. While some efforts are made by the hospital’s research department and quality team, respondents noted that leadership initiatives often lack momentum and focus.

“Leadership, including the Health Bureau, often lacks motivation to support EBP initiatives, frequently citing a lack of funds.” (FGD DATA: 1:134 ¶ 143)

“Another challenge lies within the leadership and management system. While the hospital has a research department and a quality team, there are gaps in initiating quality projects.” (FGD DATA: 1:109 ¶ 117).

## **Sub-theme 4.2: Resource and Financial Constraints**

A recurring barrier was the shortage of resources, including financial, human, and informational. Limited budgets hinder access to journals, internet services, and necessary materials for implementing EBP. Human resource shortages, particularly of senior medical staff, exacerbate the problem, reducing the hospital's capacity to support and sustain EBP initiatives effectively.

“We need funds for internet access to find reliable literature and access journals, many of which are expensive.” (FGD DATA: 1:131 ¶ 140)

“Patients may need to be seen by senior doctors, but there may not be enough senior staff available to meet this need.” (FGD DATA: 1:119 ¶ 124)

## **Sub-theme 4.3: Cultural and Behavioral Barriers**

The lack of a robust culture of research and evidence application was cited as another key challenge. Respondents noted that both healthcare professionals and institutions often undervalue the importance of research and its practical application in clinical settings.

“There is a cultural challenge, as our habit of conducting research and applying its findings is generally low.” (FGD DATA: 1:133 ¶ 142)

## **Sub-theme 4.4: Weak Monitoring and Evaluation Systems**

Although some monitoring systems exist, respondents emphasized that they are insufficient to sustain EBP adherence. These systems often fail to provide the support necessary for identifying gaps and implementing improvements from the bottom up.

“It’s difficult to identify these gaps and find solutions from the bottom up.” (FGD DATA: 1:110 ¶ 117)

“While monitoring exists, it is not strong enough.” (FGD DATA: 1:120 ¶ 125)

**Figure 8** illustrates how these barriers interconnect and ultimately hinder adherence to evidence-based practice

Figure 8: Barriers and linkage to EBP adherence

# Theme 5: Environmental Support for EBP

The respondents overwhelmingly emphasized the importance of a supportive environment in the successful implementation of EBP. A well-structured setting with adequate resources was seen as pivotal, with various positive and negative factors either facilitating or hindering EBP.

## **Sub-theme 5.1: Positive Influences**

a. **Collaboration and Communication**

Collaboration and effective communication were frequently identified as significant enablers of EBP adherence. Respondents highlighted the importance of cohesive teamwork, peer support, and multidisciplinary communication in fostering a supportive environment. Practices such as feedback processes, collaborative action plans, and continuous monitoring were noted as essential for achieving EBP goals.

“To ensure we apply EBP effectively, we need to work as a cohesive quality team and address these barriers.” (FGD DATA: 1:58 ¶ 55)

“When staff members work well together, EBP adherence is strengthened, and everyone is working toward the same objectives.” (FGD DATA: 1:98 ¶ 107)

“Having a peer group that shares knowledge and supports each other in EBP implementation strengthens the process.” (FGD DATA: 1:130 ¶ 138)

Multidisciplinary communication and the involvement of subject matter experts were also emphasized as critical for creating a supportive environment.

“To foster a supportive environment, effective multidisciplinary communication and involvement are essential.” (FGD DATA: 1:167 ¶ 158)

b. **Structured Policies and Guidelines**

Respondents emphasized the vital role of structured policies and regularly updated guidelines in fostering a positive EBP culture. Standardized protocols were seen as foundational to aligning practices with evidence-based standards.

“The hospital environment is inherently supportive of EBP due to the presence of well-established policies that guide our practice.” (FGD DATA: 1:173 ¶ 165)

The proactive involvement of hospital leadership was also identified as crucial. Leadership support, regular follow-ups on policy adherence, and resource allocation were seen as pivotal to fostering an environment conducive to EBP.

“Leadership support is crucial; when leaders endorse and support the integration of EBP, it positively impacts the willingness and capability of staff to follow through with evidence-based protocols.” (FGD DATA: 1:90 ¶ 99)

c. **Continuous Improvement Initiatives**

Respondents highlighted the hospital’s commitment to continuous quality improvement as a major facilitator of EBP. Structured policies and initiatives were instrumental in integrating EBP into daily clinical practices, ultimately improving the quality of care.

“The hospital's activities foster a culture of continuous improvement and high-quality treatment by supporting the incorporation of EBP into day-to-day activities.” (FGD DATA: 1:176)

d. **Data Accessibility**

The availability and effective management of data were consistently identified as critical facilitators of EBP adoption. Accessible data enable the translation of research findings into practical applications, ensuring that hospital practices are aligned with evidence-based standards.

“The availability of data encourages the adoption of EBP, as it facilitates the transition from findings to practical applications.” (FGD DATA: 1:185)

e. **Financial and Resource Support**

Adequate financial resources and clear budget allocations were emphasized as essential for EBP implementation. Respondents noted that sufficient funding allows healthcare professionals to work without resource-related hindrances.

“Adequate funding and resource allocation play a crucial role in promoting EBP, as it allows healthcare professionals to work without resource-related hindrances.” (FGD DATA: 1:139 ¶ 212)

## **Sub-theme 5.2: Negative Influences**

Respondents identified several challenges within the communication culture as negative influences impeding the adoption of Evidence-Based Practice (EBP). These challenges include personal biases, difficulty in aligning perspectives, and a communication environment where criticism sometimes outweighs constructive feedback. These factors hinder the establishment of a unified approach to EBP adherence. Additionally, respondents highlighted the lack of structured support and clearly defined roles within communication channels as barriers to creating the collaborative environment essential for EBP.

"Personal perspectives can create barriers when trying to share and implement evidence-based practices." (FGD Data: 1:135 ¶ 144)

"Our current communication culture presents challenges. It is sometimes difficult to promote constructive feedback over criticism." (FGD Data: 1:169 ¶ 160)

Despite some positive structures in place, respondents also noted several challenges within the healthcare environment that hinder the full adoption of EBP. These include resistance from healthcare professionals due to attitudinal barriers and skepticism about the benefits of EBP.

"One major barrier is resistance from professionals, as there can be active skepticism about the relevance or practicality of evidence-based methods." (FGD Data: 1:108 ¶ 117)

Furthermore, resource constraints, including limited manpower and occasional gaps in management support, were perceived as obstacles that undermine EBP initiatives. These challenges often delay or complicate the consistent implementation of EBP policies.

"Staff commitment, teamwork, and proper monitoring are lacking, which impacts our ability to consistently follow EBP guidelines." (FGD Data: 1:101 ¶ 111)

a. **Resource and Financial Constraints**

Respondents frequently mentioned resource and financial constraints as significant barriers to EBP implementation. Limitations in funding, human resources, and internet connectivity hinder access to updated research and literature, which are crucial for maintaining evidence-based practices.

"There are resource shortages, including a lack of human resources. For example, we need funds for internet access to find reliable literature and access evidence-based information." (FGD Data: 1:119)

Additionally, some respondents felt that policies supporting EBP could inadvertently hinder innovation. Strict adherence to established frameworks was seen as limiting creative approaches to problem-solving.

"The policies in place can inadvertently stifle innovation and hinder the progress of evidence-based practices." (FGD Data: 1:190)

b. **Inconsistent Policy Adherence**

Although policies for EBP are in place, some respondents felt there was a lack of consistent follow-up to ensure these policies are adhered to effectively.

"Regular follow-up on these policies indicates a commitment to EBP, but inconsistencies in adherence can limit their impact." (FGD Data: 1:180 ¶ 172)

c. **Staff Commitment Variability**

While many healthcare professionals are committed to EBP, attitudes toward it vary. Inconsistent commitment among staff can hinder the cohesive implementation of EBP practices.

"If the staff has a positive attitude toward EBP, its implementation becomes much more effective. However, inconsistent commitment among staff can be a barrier." (FGD Data: 1:97 ¶ 106)

d. **Data Management and Communication Issues**

Respondents also highlighted several environmental challenges that hinder EBP, such as limitations in data management capabilities and communication problems. One key issue was the limitations of the Electronic Medical Record (EMR) system, which primarily supports data entry rather than data extraction for analysis.

"Currently, the EMR system is primarily designed for data entry and recording rather than for extracting data for reporting and analysis." (FGD Data: 1:188 ¶ 181)

Moreover, some respondents noted that a top-down approach to idea acceptance could create barriers. Ideas from the executive level were sometimes difficult to implement or did not align with frontline needs. This situation was exacerbated by personal biases that impede communication and collaboration.

"Personal perspectives can create barriers when trying to share and implement EBP with colleagues. Improving communication from the top down is crucial to creating an open environment where team members feel valued and appreciated." (FGD Data: 1:169 ¶ 160)

# Theme 6: Actionable Steps for Improvement

## **Sub-theme 6.1: Action Plan Development**

Respondents identified several actionable steps to improve the implementation of Evidence-Based Practice (EBP), with a focus on developing a structured action plan. These steps emphasize data management, facilitating data availability, and ensuring continuous monitoring and improvement.

a. **Comprehensive Database Development:**

Respondents highlighted the importance of establishing a centralized database that consolidates all working reports, EBP initiatives, and quality department data. This would facilitate progress tracking and decision-making.

"Establish a centralized database that gathers all reports, EBP initiatives, and quality department data in one location. This will streamline data analysis and provide a clear overview of EBP activities across the organization." (FGD Data: 1:136)

b. **Centralized Data Analysis System:**

A centralized system for data analysis was seen as essential for monitoring EBP progress, identifying areas for improvement, and enabling evidence-based decision-making.

"Implement a centralized data analysis system to track progress, identify gaps, and make decisions based on reliable data." (FGD Data: 1:138)

c. **Data Utilization and Infrastructure Development:**

Ensuring each department has its own data management system and necessary infrastructure (e.g., internet access and library resources) was emphasized to support effective data usage.

"Access to infrastructure, such as the internet and libraries, is crucial for supporting data utilization." (FGD Data: 1:55)

d. **Regular EBP Reviews:**

Periodic EBP reviews, such as quarterly assessments, were considered vital for evaluating adherence, identifying gaps, and adjusting strategies based on new evidence.

"We conduct EBP reviews every three months to evaluate progress, identify gaps, and adapt strategies accordingly." (FGD Data: 1:66)

e. **Promoting Data-Driven Decision Making:**

Data-driven decision-making was noted as essential for advancing EBP, ensuring that decisions are informed by reliable data, which ultimately improves patient outcomes and service quality.

"EBP is about making decisions based on data. When practice is grounded in evidence, it leads to better outcomes and more informed decisions." (FGD Data: 1:51)

f. **Training and Knowledge Enhancement:**

Ongoing training programs were identified as foundational for enhancing skills and knowledge necessary for EBP integration. Respondents stressed the importance of continuous education for both leadership and staff to stay updated with evolving standards.

"Capacity-building training is essential to enhance our skills and knowledge, making it easier to adhere to EBP principles." (FGD Data: 1:115)

g. **Continuous Professional Development (CPD):**

CPD programs were highlighted as a way to engage staff, track progress, and ensure that EBP becomes part of daily practice.

"CPD programs help engage staff in EBP, track their progress, and ensure integration of EBP into daily practices." (FGD Data: 1:75)

h. **Management Support and Monitoring:**

Strong management support was considered vital for embedding EBP into the organization's culture. Regular monitoring and evaluation mechanisms were emphasized to ensure continuous improvement of EBP practices.

"Providing training for all healthcare professionals and quality officers, along with strong management support, helps embed EBP into daily routines and improves overall performance." (FGD Data: 1:109)

i. **Orientation for Key Personnel:**

Orientation programs for key personnel responsible for monitoring EBP were seen as essential for aligning everyone with EBP goals.

"Chapter owners and those responsible for monitoring EBP should receive adequate orientation to understand the issues at hand and the goals they are working toward." (FGD Data: 1:99)

j. **Promoting a Culture of Inquiry and Innovation:**

Encouraging a culture of research, innovation, and continuous improvement through evidence-based methods was identified as crucial for the successful adoption of EBP.

"Training programs should focus on increasing knowledge about EBP, particularly by encouraging staff to engage in research and apply their findings, fostering a culture of inquiry and innovation." (FGD Data: 1:214)

k. **Leadership Support:**

Leadership support was consistently noted as critical for the success of EBP implementation. Effective leadership ensures the allocation of necessary resources, such as training and continuous engagement, to address practice gaps. Integrating EBP into job descriptions and work plans ensures it becomes part of daily operations and facilitates follow-up.

"EBP initiatives should be integrated into job descriptions and work plans, ensuring that they are a core part of everyday operations and followed up on effectively." (FGD Data: 1:191)

l. **Reward Systems for Motivation:**

To motivate staff and encourage adherence to EBP, respondents suggested implementing reward systems to recognize efforts and foster a culture of accountability.

"Reward systems should be implemented to recognize staff who adhere to EBP guidelines, promoting a culture of commitment and accountability." (FGD Data: 1:198)

**Figure 9** illustrates the suggested action plan and its key linkages that aim to improve adherence to EBP across various levels of the health system.

Figure 9: suggested action plan to improve EBP
