# Supplementary material for: Exploring facilitators and barriers to adherence to evidence-based practice among health professionals at Tirunesh Beijing General Hospital, Addis Ababa, Ethiopia: a qualitative study
Source: BMC Health Serv Res. 2026 Jul 10;26:1194. doi: 10.1186/s12913-026-14923-2 (PMC13523195; doi:10.1186/s12913-026-14923-2)
Supplement: Supplementary file 2 [file 12913_2026_14923_MOESM2_ESM.docx]

**COREQ 32-Item Checklist**

# Domain 1: Research team and reflexivity

| S.No | Item | Response |
| --- | --- | --- |
| 1. | Interviewer/facilitator | The first author conducted all interviews and the focus group discussion. |
| 2. | Credentials | GMPH (Master of Public Health and BSC in Nursing) |
| 3. | Occupation | Health professional and researcher |
| 4. | Gender | Female |
| 5. | Experience and training | Trained in qualitative research methods and data collection. |
| 6. | Relationship established | No prior relationship was established with participants before the study. |
| 7. | Participant knowledge of interviewer | Participants were informed about the academic purpose of the study. |
| 8. | Interviewer characteristics | The interviewer had clinical experience; reflexivity was applied to minimize bias. |

# Domain 2: Study design

**Theoretical framework**

| No | Item | Response |
| --- | --- | --- |
| 9. | Methodological orientation | Phenomenology |

**Participant selection**

| No | Item | Response |
| --- | --- | --- |
| 10. | Sampling | Purposive sampling |
| 11. | Method of approach | Face-to-face recruitment |
| 12. | Sample size | 27 key informant interviews and 1 focus group discussion (6 participants) |
| 13. | Non-participation | No refusals or dropouts were reported. |

**Setting**

| No | Item | Response |
| --- | --- | --- |
| 14. | Setting of data collection | Tirunesh Beijing General Hospital |
| 15. | Presence of non-participants | No non-participants were present during interviews/FGD. |
| 16. | Description of sample | Health professionals including physicians, nurses, pharmacists, laboratory professionals, and health officers. |

**Data collection**

| No | Item | Response |
| --- | --- | --- |
| 17. | Interview guide | Semi-structured interview guide was used and pretested. |
| 18. | Repeat interviews | No repeat interviews were conducted. |
| 19. | Audio/visual recording | Audio recording was used with participant consent. |
| 20. | Field notes | Field notes were taken during and after interviews. |
| 21. | Duration | Each interview lasted approximately 30–60 minutes. |
| 22. | Data saturation | Data saturation was achieved. |
| 23. | Transcripts returned | Transcripts were not returned to participants. |

# Domain 3: Analysis and findings

**Data analysis**

| No | Item | Response |
| --- | --- | --- |
| 24. | Number of data coders | One primary coder; findings reviewed by the research team. |
| 25. | Coding description | Codes and themes were developed inductively from the data. |
| 26. | Derivation of themes | Themes were derived from the data using thematic analysis. |
| 27. | Software | ATLAS.ti version 9 |
| 28. | Participant checking | Member checking was conducted. |

**Reporting**

| No | Item | Response |
| --- | --- | --- |
| 29. | Quotations presented | Yes, participant quotations were included and anonymized. |
| 30. | Data and findings consistency | Yes, findings were consistent with the data presented. |
| 31. | Clarity of major themes | Major themes were clearly presented in the findings section. |
| 32. | Clarity of minor themes | Minor and divergent views were also included. |
